# Profile of deaths and causes of mortality among international migrants in Brazil, 2011–2022

**DOI:** 10.11606/s1518-8787.2025059006519

**Published:** 2026-01-12

**Authors:** João Roberto Cavalcante, Anete Trajman, Eduardo Faerstein

**Affiliations:** I Universidade do Estado do Rio de Janeiro. Instituto de Medicina Social Hesio Cordeiro. Rio de Janeiro, RJ, Brazil; II Universidade Federal do Rio de Janeiro. Departamento de Clínica Médica. Rio de Janeiro, RJ, Brazil; III McGill University. McGill International TB Centre. Montreal, Canada; IV Rede Brasileira de Pesquisa em Tuberculose. Rio de Janeiro, RJ, Brazil

**Keywords:** Migrants, Mortality, Epidemiology, Global Health, Brazil

## Abstract

**OBJECTIVE:**

To describe the mortality profile and analyze the causes of death among international migrants residing in Brazil between 2011 and 2022.

**METHODS:**

This is a cross-sectional, descriptive, and ecological study based on secondary data. The sociodemographic profile of deaths reported from 2011 to 2022 was analyzed. Absolute and relative frequencies, as well as mortality rates per 100,000 inhabitants, were calculated by country of birth, macroregion, and federative unit of residence. The underlying causes of death were also analyzed.

**RESULTS:**

A total of 173,807 deaths among international migrants were recorded between 2011 and 2022, with the highest number in 2021 (17,779; 10.2%). The predominant mortality profile was male (97,053; 55.8%), aged ≥ 81 years (104,308; 60.0%), White (136,835; 78.7%), and widowed (72,156; 41.5%). Most deceased migrants were born in Portugal (64,909; 37.3%), Japan (22,748; 13.1%), Italy (16,178; 9.3%), and Spain (12,835; 7.3%). The highest mortality rates were observed among migrants born in Lithuania (190,079/100,000), Serbia (146,794/100,000), and Hungary (96,395/100,000). The Southeast (137,457; 79.1%) and South (18,603; 10.7%) macroregions accounted for the majority of deaths, with the highest mortality rates observed in the states of Rio de Janeiro (25,349/100,000), São Paulo (23,574/100,000), and Mato Grosso do Sul (18,683/100,000). The covid-19 pandemic led to an increase in infectious and parasitic diseases from 2020 onward, peaking in 2021 (4,460; 25.0% of deaths that year). The most frequent underlying cause of death during the study period was unspecified acute myocardial infarction (12,141; 7.0%).

**CONCLUSION:**

This study highlights the need for targeted health actions addressing international migrant profiles at higher risk of death and their specific causes of mortality. However, the general invisibility of this population in public health indicators hinders the implementation of effective strategies. Policies ensuring equitable access to healthcare services, medications, and vaccines are essential to improve long-term health outcomes of this population.

## INTRODUCTION

International migration can be defined as the movement of people from their countries of origin to reside temporarily or permanently in another country^1^. This process may be driven by economic, social, or political factors^1^. The integration of international migrants into destination countries is complex in several aspects, especially regarding human rights and public policies^1^.

This phenomenon has been increasing in recent years, both in Brazil and in other countries. The most recent records indicate there were 281 million international migrants worldwide as of 2020, with 1.6 million residing in Brazil^2^. The country has historically received significant migratory flows, mainly from Portugal, Japan, Italy, and Germany, as well as enslaved people forcibly brought from African countries such as Nigeria, Côte d’Ivoire, Congo, Angola, and Mozambique^3^. Over the past 15 years, Brazil has experienced a considerable increase in migration among vulnerable populations from South and Central American countries, particularly Venezuelan and Haitian migrants^2^.

In the field of health, international migrants are often in situations of vulnerability^4^. Depending on their country of origin, the health conditions of this group are a matter of concern, as they are often victims of violence and/or exposed to infectious diseases before and during international migration^4^. These more vulnerable populations may have untreated health conditions and complications, such as mental and psychosocial distress, as well as noncommunicable chronic diseases^4,5^. Language barriers, lack of knowledge about local health systems, and stress associated with the migration process all contribute to this scenario^4^.

In Brazil, the lack of specific data on the health of these populations creates challenges for the development of effective public policies^6^. The *Sistema de Informações sobre Mortalidade* (SIM – Brazilian Mortality Information System) potentially represents an essential tool to address this information gap, as it includes the country of birth of deceased individuals^7^. However, although SIM data have been available since 1979, there are no known analyses or publications of this information at the national level to date, possibly due to the need to calculate denominators for mortality coefficients (MC), which would enable comparisons^8^.

Analyzing and describing these causes among international migrants residing in Brazil is essential for the development of health management strategies towards reducing the risk of deaths from preventable causes and improving access to healthcare services for this population. Thus, this study aims to describe the mortality profile and analyze the causes of death among international migrants residing in Brazil from 2011 to 2022.

## METHODS

This is a cross-sectional, descriptive, and ecological study based on secondary data. The units of analysis were countries of birth, federative units (FUs), macroregions of residence in Brazil, and underlying causes of death.

Brazil is composed of 5,570 municipalities, organized into 26 states and one Federal District, forming five major macroregions^9^. With a total area of 8,515,767 km^2^, it is the fifth largest country in the world in terms of territorial extension^9^. In 2022, the country had an estimated population of 203 million inhabitants, ranking as the seventh most populous country in the world, with a population density of approximately 25.2 inhabitants per km^2 9^.

All recorded deaths of international migrants from 2011 to 2022 were included. Year 2023 was not considered because the available data were incomplete at the time the databases were obtained. To distinguish international migrants from native-born Brazilians in the SIM databases, the variable “NATURAL” was used, which presents good completeness, since information about place of birth is mandatory in death certificates^7^. This variable, recorded in the SIM database, is publicly available on the OpenDATASUS website of the Brazilian Ministry of Health^7^.

Absolute and relative frequencies were analyzed for year of death, sex, age group, race/skin color, marital status, ICD-10 chapter, and underlying cause of death^10^. To calculate MC per 100,000 inhabitants, it was necessary to elaborate denominators representing the population of migrants residing in Brazil, by country of birth, FU, and macroregion during the study period. Open data from the 2010 Demographic Census of the *Instituto Brasileiro de Geografia e Estatítica* and from the *Sistema de Registro Nacional Migratório* (SISMIGRA – National Migration Registration System) of the Ministry of Justice (2011–2022) were used, along with mortality data from SIM (2011–2022)^7,11,12^. The SISMIGRA database compiles the number of international migrants with active registration in the country. The completeness of variables in these systems is high, since the variable identifying whether an individual is an international migrant is mandatory^7,11,12^. All SISMIGRA categories (resident, temporary, provisional, and border migrant) were considered^12^. Using these data, it was possible to estimate, for the first time, the populations of international migrants residing in Brazil from 2011 to 2022. The formula used to calculate these populations for 2011 was:


Y=A+B−C

Y = estimated population of international migrants residing in Brazil by country of birth, macroregion, or FU (2011);

A = number of international migrants residing in Brazil by country of birth, macroregion, or FU in 2010 (2010 Census);

B = number of international migrants who entered Brazil by country of birth, macroregion, or FU in 2011 (Sismigra, 2011);

C = number of deaths among international migrants in Brazil by country of birth, macroregion, or FU in 2011 (SIM, 2011).

For populations from 2012 to 2022, it was no longer necessary to use the 2010 Census separately, as it was already incorporated into the population estimated for 2011. The formula used to calculate the populations for subsequent years was:


Y=A+B−C

Y = estimated population of international migrants residing in Brazil by country of birth, macroregion, or FU in the given year;

A = number of international migrants residing in Brazil by country of birth, macroregion, or FU in the previous year (calculated by the authors);

B = number of international migrants who entered Brazil by country of birth, macroregion, or FU in the given year (Sismigra);

C = number of deaths among international migrants in Brazil by country of birth, macroregion, or FU in the given year (SIM).

The mid-year population was used in the denominator of the MC^a^, with July 2 being considered the midpoint of the period of each year^13^, as shown in the following formula:


$$MC=\frac{\text{ Number of deaths }}{\text{ Mid-year population }}\times 100,000$$

During the analysis, it was necessary to explore the main causes of death among migrants from specific countries that have faced or are currently facing migration crises, or that have vulnerable migrant populations residing in Brazil in recent years, namely: Angola, Haiti, Syria, and Venezuela^14^.

The RStudio (version 3.3.0+) software was used for data processing and analysis. As this study used secondary, aggregated, and publicly available data, ethical approval was not required, according to Resolution No. 510 of 2016 of the Brazilian National Council for Ethics in Research. The databases analyzed were public and anonymized, as established under the General Data Protection Law (Law No. 13,709/2018)^15,16^.

## RESULTS

Between 2011 and 2022, a total of 173,807 deaths among international migrants residing in Brazil were recorded, with the highest number occurring in 2021 (Table 1). Most deaths occurred among older males, with a mean age of 79.8 years. The predominant race/skin color was White, followed by Asian. However, in 2021, the proportion of mixed-race individuals surpassed that of Asians. The highest frequency of deaths was observed among widowed individuals, followed by married ones (Table 1).

**Table 1:** Sociodemographic profile of deaths among international migrants residing in Brazil, 2011–2022.

Sociodemographic characteristics	2011		2012		2013		2014		2015		2016		2017		2018		2019		2020		2021		2022		Total	
	n	%	n	%	n	%	n	%	n	%	n	%	n	%	n	%	n	%	n	%	n	%	n	%	n	%
Year of death	13,730	7.9	13,620	7.8	14,099	8.1	14,262	8.2	14,258	8.2	14,227	8.2	13,843	8.0	13,850	8.0	14,209	8.2	15,316	8.8	17,779	10.2	14,614	8.4	173,807	100
Sex																										
Male	7,636	55.6	7,580	55.7	7,904	56.1	7,871	55.2	7,770	54.5	7,887	55.4	7,660	55.3	7,608	54.9	7,921	55.7	8,823	57.6	10,349	58.2	8,044	55.0	97,053	55.8
Female	6,094	44.4	6,039	44.3	6,194	43.9	6,391	44.8	6,488	45.5	6,339	44.6	6,183	44.7	6,241	45.1	6,288	44.3	6,488	42.4	7,427	41.8	6,563	44.9	76,735	44.1
Ignored	0	0	1	0	1	0	0	0	0	0	1	0	0	0	1	0	0	0	5	0	3	0	7	0	19	0
Age group (years)																										
< 1	12	0.1	11	0.1	7	0	9	0.1	5	0	10	0.1	10	0.1	14	0.1	23	0.2	13	0.1	39	0.2	19	0.1	172	0.1
1–10	29	0.2	24	0.2	26	0.2	28	0.2	22	0.2	21	0.1	23	0.2	35	0.3	57	0.4	36	0.2	50	0.3	59	0.4	410	0.2
11–20	53	0.4	52	0.4	53	0.4	40	0.3	42	0.3	45	0.3	40	0.3	68	0.5	70	0.5	89	0.6	95	0.5	125	0.9	772	0.4
21–30	80	0.6	96	0.7	106	0.8	121	0.8	122	0.9	138	1.0	156	1.1	197	1.4	189	1.3	257	1.7	339	1.9	290	2.0	2,091	1.2
31–40	114	0.8	102	0.7	100	0.7	133	0.9	164	1.2	164	1.2	172	1.2	212	1.5	239	1.6	347	2.3	437	2.5	367	2.5	2,551	1.5
41–50	174	1.3	185	1.4	182	1.3	220	1.5	209	1.5	229	1.6	201	1.5	235	1.7	292	2.0	377	2.5	637	3.6	407	2.8	3,348	1.9
51–60	582	4.2	498	3.7	510	3.6	473	3.3	461	3.2	462	3.2	432	3.1	470	3.4	465	3.1	582	3.8	899	5.1	599	4.1	6,433	3.7
61–70	1,409	10.3	1,417	10.4	1,490	10.6	1,503	10.5	1,453	10.2	1,487	10.5	1,388	10.0	1,357	9.8	1,375	9.3	1,589	10.4	1,941	10.9	1,323	9.1	17,732	10.2
71–80	3,211	23.4	3,096	22.7	3,036	21.5	3,025	21.2	2,862	20.1	2,796	19.7	2,710	19.6	2,651	19.1	2,746	18.5	3,189	20.8	3,801	21.4	2,795	19.1	35,918	20.7
≥ 81	8,063	58.7	8,135	59.7	8,588	60.9	8,707	61.1	8,916	62.5	8,873	62.4	8,708	62.9	8,607	62.1	8,745	58.9	8,833	57.7	9,537	53.6	8,596	58.8	104,308	60.0
Ignored	3	0	4	0	1	0	3	0	2	0	2	0	3	0	4	0	8	0.1	4	0	4	0	34	0.2	72	0
Race/skin color																										
White	11,129	81.1	10,908	80.1	11,388	80.8	11,520	80.8	11,479	80.5	11,332	79.7	11,116	80.3	10,976	79.2	11,160	78.5	11,604	75.8	13,274	74.7	10,949	74.9	136,835	78.7
Black	95	0.7	96	0.7	117	0.8	164	1.1	183	1.3	200	1.4	201	1.5	265	1.9	269	1.9	428	2.8	484	2.7	380	2.6	2,882	1.7
Asian	1,674	12.2	1,721	12.6	1,736	12.3	1,743	12.2	1,740	12.2	1,812	12.7	1,668	12.0	1,565	11.3	1,494	10.5	1,656	10.8	1,776	10.0	1,519	10.4	20,104	11.6
Mixed-race	424	3.1	463	3.4	477	3.4	515	3.6	626	4.4	638	4.5	630	4.6	780	5.6	992	7.0	1,348	8.8	1,912	10.8	1,491	10.2	10,296	5.9
Indigenous	14	0.1	15	0.1	6	0	19	0.1	8	0.1	19	0.1	30	0.2	36	0.3	53	0.4	62	0.4	54	0.3	70	0.5	386	0.2
Ignored	394	2.9	417	3.1	375	2.7	301	2.1	222	1.6	226	1.6	198	1.4	228	1.6	241	1.7	218	1.4	279	1.6	205	1.4	3,304	1.9
Marital status																										
Single	1,323	9.6	1,278	9.4	1,378	9.8	1,417	9.9	1,407	9.9	1,441	10.1	1,404	10.1	1,554	11.2	1,705	12.0	1,998	13.0	2,465	13.9	2,173	14.9	19,543	11.2
Married	5,371	39.1	5,269	38.7	5,361	38.0	5,381	37.7	5,369	37.7	5,355	37.6	5,129	37.1	4,924	35.6	5,047	35.5	5,567	36.3	6,598	37.1	4,825	33.0	64,196	36.9
Widowed	5,887	42.9	5,836	42.8	6,069	43.0	6,198	43.5	6,201	43.5	6,056	42.6	5,970	43.1	5,908	42.7	5,895	41.5	5,867	38.3	6,457	36.3	5,812	39.8	72,156	41.5
Separated/divorced	610	4.4	655	4.8	657	4.7	679	4.8	673	4.7	745	5.2	764	5.5	812	5.9	840	5.9	952	6.2	1,074	6.0	906	6.2	9,367	5.4
Common-law marriage	107	0.8	161	1.2	139	1.0	159	1.1	169	1.2	198	1.4	198	1.4	248	1.8	251	1.8	348	2.3	403	2.3	276	1.9	2,657	1.5
Ignored	432	3.1	421	3.1	495	3.5	428	3.0	439	3.1	432	3.0	378	2.7	404	2.9	471	3.3	584	3.8	782	4.4	622	4.3	5,888	3.4

Most international migrants who died were born in Portugal, followed by Japan, Italy, Spain, Germany, Argentina, Bolivia, Paraguay, Lebanon, and Uruguay (Table 2). The highest MC during the study period were observed among international migrants born in Lithuania, followed by Serbia, Hungary, Poland, Jordan, Japan, Portugal, Romania, Egypt, and Lebanon (Table 2).

**Table 2:** Mortality among international migrants residing in Brazil by country of birth, 2011–2022.

Country of birth	2011		2012		2013		2014		2015		2016		2017		2018		2019		2020		2021		2022		Full study period (2011–2022)	
	n (%)	MC	n (%)	MC	n (%)	MC	n (%)	MC	n (%)	MC	n (%)	MC	n (%)	MC	n (%)	MC	n (%)	MC	n (%)	MC	n (%)	MC	n (%)	MC	n (%)	MC
South Africa	10 (0.1)	712.8	8 (0.1)	530.9	10 (0.1)	571.8	9 (0.1)	451.6	15 (0.1)	682.6	11 (0.1)	465.0	8 (0.1)	322.5	11 (0.1)	428.1	15 (0.1)	567.9	11 (0.1)	408.5	13 (0.1)	473.2	9 (0.1)	320.1	130 (0.1)	5,364.7
Germany	403 (2.9)	3,014.2	393 (2.9)	2,732.7	432 (3.1)	2,608.1	414 (2.9)	2,184.0	423 (3.0)	1,991.5	379 (2.7)	1,630.7	385 (2.8)	1,548.2	401 (2.9)	1,538.3	433 (3.0)	1,602.5	336 (2.2)	1,221.6	702 (3.9)	2,552.2	576 (3.9)	2,080.7	5,277 (3.0)	21,937.7
Angola	42 (0.3)	683.9	44 (0.3)	664.8	38 (0.3)	496.1	45 (0.3)	3.6	68 (0.5)	640.1	58 (0.4)	465.2	71 (0.5)	513.1	72 (0.5)	489.6	67 (0.5)	433.9	62 (0.4)	388.4	92 (0.5)	546.5	61 (0.4)	328.0	720 (0.4)	5,474.1
Argentina	250 (1.8)	981.9	292 (2.1)	1,061.4	300 (2.1)	933.2	291 (2.0)	770.6	280 (2.0)	648.0	318 (2.2)	663.9	342 (2.5)	656.1	360 (2.6)	640.8	364 (2.6)	598.6	362 (2.4)	560.7	475 (2.7)	705.5	392 (2.7)	549.3	4,026 (2.3)	8,050.4
Australia	22 (0.2)	2,973.0	20 (0.1)	2,372.5	7 (0)	663.2	11 (0.1)	846.2	9 (0.1)	578.0	11 (0.1)	608.9	7 (0.1)	349.5	1 (0)	46.9	8 (0.1)	356.3	11 (0.1)	472.3	3 (0)	125.8	2 (0)	81.5	112 (0.1)	5,880.0
Austria	71 (0.5)	4,159.3	64 (0.5)	3,619.9	64 (0.5)	3,359.6	83 (0.6)	4,037.0	52 (0.4)	2,335.5	63 (0.4)	2,624.5	64 (0.5)	2,529.1	63 (0.5)	2,407.3	64 (0.5)	2,391.2	64 (0.4)	2,378.7	62 (0.3)	2,299.3	52 (0.4)	1,893.7	766 (0.4)	31,068.7
Belgium	22 (0.2)	1,406.6	26 (0.2)	1,496.4	27 (0.2)	1,279.3	34 (0.2)	1,347.9	31 (0.2)	1,060.2	29 (0.2)	888.8	26 (0.2)	731.6	36 (0.3)	945.9	44 (0.3)	1,095.9	28 (0.2)	675.8	55 (0.3)	1,308.9	41 (0.3)	956.3	399 (0.2)	11,706.0
Bolivia	197 (1.4)	464.7	189 (1.4)	394.0	218 (1.5)	367.4	242 (1.7)	352.8	262 (1.8)	349.3	273 (1.9)	339.2	225 (1.6)	261.3	293 (2.1)	3.6	328 (2.3)	325.1	504 (3.3)	477.2	657 (3.7)	598.5	453 (3.1)	392.5	3,841 (2.2)	4,611.1
Cape Verde	27 (0.2)	2,124.3	5 (0)	359.2	11 (0.1)	664.5	7 (0)	353.1	7 (0)	302.6	1 (0)	39.1	8 (0.1)	292.0	10 (0.1)	347.3	7 (0)	231.9	9 (0.1)	288.4	10 (0.1)	317.0	10 (0.1)	312.6	112 (0.1)	4,227.6
Chile	113 (0.8)	827.2	127 (0.9)	890.2	134 (1.0)	864.0	158 (1.1)	938.3	142 (1.0)	782.9	154 (1.1)	797.7	175 (1.3)	859.4	185 (1.3)	868.2	187 (1.3)	841.3	263 (1.7)	1,149.2	327 (1.8)	1,403.0	233 (1.6)	977.1	2,198 (1.3)	11,081.7
China	148 (1.1)	819.1	167 (1.2)	832.6	157 (1.1)	643.0	174 (1.2)	590.8	163 (1.1)	474.4	187 (1.3)	485.1	174 (1.3)	409.6	200 (1.4)	432.9	186 (1.3)	377.7	223 (1.5)	439.5	351 (2.0)	685.7	228 (1.6)	439.5	2,358 (1.4)	5,820.5
Colombia	20 (0.1)	262.2	33 (0.2)	349.0	26 (0.2)	176.1	38 (0.3)	174.5	50 (0.4)	172.5	57 (0.4)	155.8	48 (0.3)	106.6	53 (0.4)	97.4	72 (0.5)	113.3	103 (0.7)	148.4	157 (0.9)	214.5	102 (0.7)	131.4	759 (0.4)	1,859.9
South Korea	83 (0.6)	976.1	76 (0.6)	820.4	80 (0.6)	744.1	75 (0.5)	602.7	77 (0.5)	525.6	97 (0.7)	584.7	107 (0.8)	605.7	90 (0.6)	489.4	117 (0.8)	613.3	118 (0.8)	602.5	139 (0.8)	695.5	107 (0.7)	524.8	1,166 (0.7)	6,808.0
Cuba	11 (0.1)	627.9	12 (0.1)	634.8	12 (0.1)	246.1	16 (0.1)	141.9	22 (0.2)	138.4	28 (0.2)	145.6	26 (0.2)	107.2	22 (0.2)	78.5	24 (0.2)	80.2	46 (0.3)	148.2	59 (0.3)	185.9	35 (0.2)	103.8	313 (0.2)	1,439.8
Egypt	75 (0.5)	6,097.6	80 (0.6)	6,184.8	77 (0.5)	5,565.6	69 (0.5)	4,760.3	97 (0.7)	6,419.6	76 (0.5)	4,772.4	88 (0.6)	5,114.8	75 (0.5)	4,088.3	67 (0.5)	3,471.5	80 (0.5)	4,006.0	121 (0.7)	6,025.9	87 (0.6)	4,288.9	992 (0.6)	59,885.3
Ecuador	16 (0.1)	1,009.5	13 (0.1)	720.6	5 (0)	215.5	9 (0.1)	298.1	8 (0.1)	204.7	5 (0)	100.3	7 (0.1)	115.3	11 (0.1)	156.3	7 (0)	87.9	16 (0.1)	186.0	14 (0.1)	153.9	9 (0.1)	90.2	120 (0.1)	2,170.3
Spain	1,152 (8.4)	4,524.7	1,124 (8.3)	4,272.0	1,155 (8.2)	4,050.6	1,062 (7.4)	3,413.0	1,081 (7.6)	3,246.2	1,101 (7.7)	3,155.8	1,013 (7.3)	2,832.7	1,010 (7.3)	2,807.0	1,018 (7.2)	2,836.8	1,057 (6.9)	2,983.6.	1,088 (6.1)	3,130.5	974 (6.7)	2,843.5	12,835 (7.4)	36,334.3
USA	73 (0.5)	385.3	95 (0.7)	444.7	78 (0.6)	294.3	91 (0.6)	285.7	93 (0.7)	254.8	94 (0.7)	233.4	99 (0.7)	227.7	106 (0.8)	228.5	97 (0.7)	196.4	119 (0.8)	231.8	123 (0.7)	232.9	118 (0.8)	214.9	1,186 (0.7)	2,832.4
France	133 (1.0)	1,368.7	129 (0.9)	1,170.8	119 (0.8)	848.8	128 (0.9)	735.1	96 (0.7)	464.2	117 (0.8)	494.8	125 (0.9)	474.3	117 (0.8)	406.0	119 (0.8)	382.7	119 (0.8)	365.2	173 (1.0)	518.0	126 (0.9)	363.4	1,501 (0.9)	6,003.9
Greece	55 (0.4)	4,696.8	54 (0.4)	4,368.9	78 (0.6)	5,577.4	79 (0.6)	5,039.9	74 (0.5)	4,202.2	69 (0.5)	3,479.6	53 (0.4)	2,412.9	62 (0.4)	2,650.1	85 (0.6)	3,619.3	67 (0.4)	2,908.0	78 (0.4)	3,457.4	62 (0.4)	2,799.7	816 (0.5)	39,047.7
Guyana	8 (0.1)	1,126.8	9 (0.1)	1,252.6	8 (0.1)	1,078.2	5 (0)	640.6	10 (0.1)	1,209.2	21 (0.1)	2,456.1	14 (0.1)	1,579.2	25 (0.2)	2,691.1	15 (0.1)	1,515.9	19 (0.1)	1,817.3	25 (0.1)	2,347.4	30 (0.2)	2,678.6	189 (0.1)	21,705.4
Haiti	1 (0)	36.0	10 (0.1)	198.1	17 (0.1)	114.6	49 (0.3)	149.7	75 (0.5)	139.2	86 (0.6)	114.2	103 (0.7)	110.4	139 (1.0)	126.3	147 (1.0)	110.3	242 (1.6)	156.5	257 (1.4)	155.4	192 (1.3)	112.9	1,318 (0.8)	1,563.7
Netherlands	46 (0.3)	1,510.2	50 (0.4)	1,480.4	52 (0.4)	1,279.4	55 (0.4)	1,132.2	59 (0.4)	1,035.0	64 (0.4)	988.4	63 (0.5)	885.5	57 (0.4)	755.6	54 (0.4)	687.9	53 (0.3)	659.7	68 (0.4)	835.5	55 (0.4)	662.0	676 (0.4)	9,948.5
Hungary	72 (0.5)	11,881.2	67 (0.5)	10,850.2	63 (0.4)	9,820.7	65 (0.5)	9,826.2	62 (0.4)	8,857.1	71 (0.5)	9,752.7	44 (0.3)	6,039.8	54 (0.4)	7,427.8	63 (0.4)	8,955.2	44 (0.3)	6,475.3	54 (0.3)	8,083.8	43 (0.3)	6,544.9	702 (0.4)	96,395.5
Indonesia	17 (0.1)	1,054.6	9 (0.1)	406.9	14 (0.1)	421.1	4 (0)	94.7	8 (0.1)	169.3	7 (0)	143.0	13 (0.1)	260.8	14 (0.1)	277.6	11 (0.1)	215.5	21 (0.1)	408.3	19 (0.1)	367.3	14 (0.1)	267.8	151 (0.1)	3,057.1
Israel	22 (0.2)	2,073.5	11 (0.1)	997.7	21 (0.1)	1,789.5	20 (0.1)	1,596.2	18 (0.1)	1,345.3	19 (0.1)	1,363.5	26 (0.2)	1,811.8	19 (0.1)	1,290.8	27 (0.2)	1,805.4	31 (0.2)	2,046.2	29 (0.2)	1,893.0	29 (0.2)	1,885.0	272 (0.2)	19,232.8
Italy	1,278 (9.3)	4,291.6	1,328 (9.8)	4,349.3	1,350 (9.6)	4,198.7	1,412 (9.9)	4,120.1	1,381 (9.7)	3,782.1	1,365 (9.6)	3,576.7	1,340 (9.7)	3,417.8	1,297 (9.4)	3,264.9	1,338 (9.4)	3,354.8	1,362 (8.9)	3,450.5	1,507 (8.5)	3,902.4	1,220 (8.3)	3,212.1	16,178 (9.3)	41,819.8
Japan	1,936 (14.1)	5,925.9	1,995 (14.6)	6,126.0	2,052 (14.6)	6,289.9	2,019 (14.2)	6,152.6	2,035 (14.3)	6,188.3	2,046 (14.4)	6,246.7	1,904 (13.7)	5,891.5	1,790 (12.9)	5,602.9	1,685 (11.9)	5,337.3	1,825 (11.9)	5,972.7	1,836 (10.3)	6,296.3	1,625 (11.1)	5,794.1	22,748 (13.1)	69,917.5
Jordan	35 (0.3)	8,046.0	28 (0.2)	6,504.1	23 (0.2)	5,386.4	35 (0.2)	7,918.6	24 (0.2)	5,122.7	34 (0.2)	7,003.1	27 (0.2)	5,373.1	26 (0.2)	4,878.0	32 (0.2)	5,844.7	28 (0.2)	5,204.5	42 (0.2)	8,022.9	22 (0.2)	4,288.5	356 (0.2)	72,064.8
Latvia	11 (0.1)	8,270.7	14 (0.1)	8,536.6	10 (0.1)	4,566.2	7 (0)	2,631.6	11 (0.1)	3,531.3	13 (0.1)	3,651.7	10 (0.1)	2,611.0	9 (0.1)	2,278.5	4 (0)	986.4	6 (0)	1,452.8	9 (0.1)	2,142.9	8 (0.1)	1,851.9	112 (0.1)	30,311.2
Lebanon	235 (1.7)	3,528.0	223 (1.6)	3,318.5	289 (2.1)	4,224.5	264 (1.9)	3,798.0	265 (1.9)	3,776.0	306 (2.2)	4,347.5	289 (2.1)	4,100.5	230 (1.7)	3,250.7	284 (2.0)	3,988.5	311 (2.0)	4,385.2	358 (2.0)	5,107.0	252 (1.7)	3,592.3	3,306 (1.9)	46,938.6
Lithuania	99 (0.7)	23,970.9	91 (0.7)	22,864.3	65 (0.5)	17,195.8	63 (0.4)	17,142.9	61 (0.4)	16,968.0	52 (0.4)	14,689.3	55 (0.4)	16,105.4	41 (0.3)	13,162.1	30 (0.2)	10,869.6	45 (0.3)	18,867.9	36 (0.2)	18,181.8	23 (0.2)	13,649.9	661 (0.4)	190,079.1
Morocco	6 (0)	1,600.0	3 (0)	714.3	12 (0.1)	2,245.1	14 (0.1)	2,054.3	13 (0.1)	1,553.2	11 (0.1)	1,092.4	13 (0.1)	1,090.6	14 (0.1)	1,003.2	11 (0.1)	681.1	18 (0.1)	1,029.2	14 (0.1)	769.2	19 (0.1)	970.9	148 (0.1)	13,460.7
Mozambique	1 (0)	83.8	11 (0.1)	810.9	8 (0.1)	471.6	9 (0.1)	421.2	8 (0.1)	311.2	13 (0.1)	449.0	14 (0.1)	436.3	11 (0.1)	313.3	10 (0.1)	262.4	21 (0.1)	521.4	18 (0.1)	431.5	19 (0.1)	431.3	143 (0.1)	4,685.1
Nigeria	8 (0.1)	583.5	6 (0)	397.0	11 (0.1)	593.3	12 (0.1)	482.2	11 (0.1)	347.2	18 (0.1)	487.2	11 (0.1)	266.8	15 (0.1)	342.6	12 (0.1)	259.5	19 (0.1)	393.1	19 (0.1)	379.2	18 (0.1)	341.1	160 (0.1)	4,093.6.
Paraguay	246 (1.8)	1,046.4	218 (1.6)	867.7	226 (1.6)	792.9	232 (1.6)	718.7	272 (1.9)	757.2	279 (2.0)	718.3	263 (1.9)	636.0	302 (2.2)	690.9	326 (2.3)	707.1	358 (2.3)	748.1	464 (2.6)	935.6	350 (2.4)	665.9	3,536 (2.0)	8,818.4
Peru	71 (0.5)	519.8	82 (0.6)	524.3	71 (0.5)	363.8	110 (0.8)	469.0	105 (0.7)	386.8	100 (0.7)	330.2	97 (0.7)	292.7	87 (0.6)	242	119 (0.8)	308.0	209 (1.4)	517.3	254 (1.4)	611.0	157 (1.1)	3.6	1,462 (0.8)	4,610.2
Poland	299 (2.2)	16,537.6	275 (2.0)	14,274.6	245 (1.7)	10,984.1	297 (2.1)	11,320.8	245 (1.7)	8,088.5	238 (1.7)	6,995.9	209 (1.5)	5,656.3	192 (1.4)	4,898.6	217 (1.5)	5,383.9	205 (1.3)	5,147.5	206 (1.2)	5,250.4	178 (1.2)	4,520.1	2,806 (1.6)	79,075.7
Portugal	5,289 (38.5)	4,835.7	5,305 (39.0)	4,871.1	5,475 (38.9)	5,059.0	5,550 (38.9)	5,171.6	5,579 (39.1)	5,292.7	5,430 (38.2)	5,294.2	5,345 (38.6)	5,396.3	5,268 (38.0)	5,538.9	5,307 (37.3)	5,838.6	5,450 (35.6)	6,317.3	5,966 (33.5)	7,354.6	4,945 (33.8)	6,475.9	64,909 (37.3)	64,389.2
United Kingdom	35 (0.3)	726.0	31 (0.2)	553.7	52 (0.4)	724.1	48 (0.3)	546.6	38 (0.3)	370.3	53 (0.4)	456.3	37 (0.3)	291.6	37 (0.3)	275.6	37 (0.3)	262.9	50 (0.3)	343.9	64 (0.4)	430.6	53 (0.4)	346.1	535 (0.3)	4,402.8
Czech Republic	21 (0.2)	6,441.7	20 (0.1)	5,730.7	18 (0.1)	4,265.4	24 (0.2)	4,678.4	29 (0.2)	4,953.0	27 (0.2)	4,208.9	24 (0.2)	3,526.8	21 (0.2)	2,935.0	20 (0.1)	2,631.6	18 (0.1)	2,282.8	12 (0.1)	1,494.4	20 (0.1)	2,428.7	254 (0.1)	38,426.6
Romania	171 (1.2)	27,099.8	141 (1.0)	18,700.3	158 (1.1)	15,520.6	148 (1.0)	11,526.5	102 (0.7)	6,484.4	110 (0.8)	5,643.9	99 (0.7)	4,395.1	84 (0.6)	3,488.4	90 (0.6)	3,608.7	66 (0.4)	2,637.9	70 (0.4)	2,815.2	61 (0.4)	2,440.5	1,300 (0.7)	61,882.7
Russia	55 (0.4)	4,293.5	52 (0.4)	3,419.9	64 (0.5)	3,203.2	53 (0.4)	2,161.1	48 (0.3)	1,647.2	41 (0.3)	1,201.6	49 (0.4)	1,277.5	31 (0.2)	740.7	28 (0.2)	620.2	37 (0.2)	775.1	40 (0.2)	785.8	19 (0.1)	337.8	517 (0.3)	14,267.0
Serbia	67 (0.5)	24,814.8	67 (0.5)	24,907.1	88 (0.6)	33,270.3	41 (0.3)	14,089.3	66 (0.5)	19,969.7	30 (0.2)	8,438.8	36 (0.3)	9,536.4	28 (0.2)	7,282.2	27 (0.2)	7,031.3	37 (0.2)	9,866.7	34 (0.2)	9,497.2	17 (0.1)	4,864.1	538 (0.3)	146,794.0
Syria	84 (0.6)	8,203.1	84 (0.6)	7,850.5	89 (0.6)	7,038.4	86 (0.6)	4,162.6	89 (0.6)	2,834.4	73 (0.5)	1,947.7	89 (0.6)	2,159.9	80 (0.6)	1,875.3	64 (0.5)	1,526.0	93 (0.6)	2,259.7	84 (0.5)	2,085.9	69 (0.5)	1,746.6	984 (0.6)	25,011.1
Switzerland	52 (0.4)	2,195.0	59 (0.4)	2,341.3	63 (0.4)	2,241.2	54 (0.4)	1,710.5	63 (0.4)	1,704.8	53 (0.4)	1,233.4	53 (0.4)	1,085.4	74 (0.5)	1,367.1	64 (0.5)	1,090.7	68 (0.4)	1,105.7	68 (0.4)	1,087.7	68 (0.5)	1,055.7	739 (0.4)	16,100.2
Türkiye	25 (0.2)	8,771.9	22 (0.2)	6,547.6	22 (0.2)	4,835.2	16 (0.1)	2,644.6	24 (0.2)	3,129.1	15 (0.1)	1,608.6	12 (0.1)	1,087.9	19 (0.1)	1,515.8	18 (0.1)	1,312.4	9 (0.1)	619.8	11 (0.1)	715.7	17 (0.1)	1,012.8	210 (0.1)	20,633.8
Ukraine	22 (0.2)	3,288.5	16 (0.1)	1,901.4	26 (0.2)	2,125.1	17 (0.1)	1,023.8	19 (0.1)	915.7	20 (0.1)	820.5	16 (0.1)	588.2	18 (0.1)	618.1	12 (0.1)	392.2	15 (0.1)	477.6	13 (0.1)	403.7	15 (0.1)	428.3	209 (0.1)	8,104.7
Uruguay	167 (1.2)	847.3	203 (1.5)	980.8	228 (1.6)	989.9	210 (1.5)	808.8	233 (1.6)	789.0	229 (1.6)	687.3	220 (1.6)	596.5	231 (1.7)	560.2	267 (1.9)	584.1	287 (1.9)	596.6	354 (2.0)	720.0	334 (2.3)	659.0	2,963 (1.7)	8,441.5
Venezuela	10 (0.1)	340.8	15 (0.1)	448.0	8 (0.1)	187.0	13 (0.1)	234.9	15 (0.1)	206.0	16 (0.1)	142.7	54 (0.4)	217.3	140 (1.0)	198.2	335 (2.4)	215.3	451 (2.9)	203.2	731 (4.1)	268.2	731 (5.0)	209.3	2,519 (1.4)	13,968.6
Others	417 (3.0)	1,558.4	223 (1.6)	665.3	234 (1.7)	483.9	289 (2.0)	444.2	241 (1.7)	293.6	257 (1.8)	265.3	272 (2.0)	250.4	319 (2.3)	267.9	247 (1.7)	192.4	304 (2.0)	226.2	436 (2.4)	315.1	340 (2.3)	234.1	3,579 (2.1)	3,483.4
Total	13,729 (100)	2,778.9	13,619 (100)	2,563.4	14,090 (100)	2,269.2	14,256 (100)	1,952.1	14,259 (100)	1,701.5	14,225 (100)	1,519.1	13,852 (100)	1,341.5	13,850 (100)	1,199.4	14,210 (100)	1,079.2	15,300 (100)	1,065.9	17,797 (100)	1,175.4	14,620 (100)	897.9	173,807 (100)	17,654.4

MC: mortality coefficient; USA: United States of America.

The MC for 2011–2022 was 17,654.7 deaths per 100,000 inhabitants. The Southeast of Brazil recorded the highest number of deaths, followed by the South. The Southeast also ranked first in MC, followed by the Midwest. The states with the highest number of deaths were São Paulo, Rio de Janeiro, and Paraná, respectively. The highest MC were observed in the states of Rio de Janeiro, São Paulo, and Mato Grosso do Sul, respectively (Table 3).

**Table 3:** Mortality among international migrants residing in Brazil by Federative Unit and macroregion, 2011–2022.

Macroregion/ Federative Unit	2011			2012			2013			2014			2015			2016			2017			2018			2019			2020			2021			2022			Total		
	n	%	MC	n	%	MC	n	%	MC	n	%	MC	n	%	MC	n	%	MC	n	%	MC	n	%	MC	n	%	MC	n	%	MC	n	%	MC	n	%	MC	n	%	MC
North	208	1.5	1,044.3	236	1.7	1,087.9	222	1.6	860.0	236	1.7	768.1	296	2.1	828.9	308	2.2	734.9	350	2.5	631.0	400	2.9	425.8	570	4.0	353.5	758	4.9	359.5	974	5.5	402.9	815	5.6	282.7	5,373	3.1	11,035.3
Acre	5	0	455.0	11	0.1	891.4	9	0.1	601.2	10	0.1	566.6	15	0.1	744.2	13	0.1	582.0	17	0.1	697.0	12	0.1	451.1	10	0.1	338.1	21	0.1	656.1	50	0.3	1,506.0	18	0.1	504.8	191	0.1	8,175.5
Amapá	1	0	130.2	4	0	460.6	8	0.1	765.9	2	0	167.2	3	0	224.6	3	0	203.3	5	0	307.7	3	0	169.2	11	0.1	568.5	9	0.1	438.4	9	0.1	420.8	9	0.1	401.2	67	0	4,321.9
Amazonas	81	0.6	914.7	85	0.6	876.5	74	0.5	625.0	90	0.6	622.0	89	0.6	525.2	99	0.7	506.1	103	0.7	448.3	95	0.7	307.6	158	1.1	330.1	255	1.7	412.6	294	1.7	427.8	233	1.6	302.2	1,656	1.0	7,786.0
Pará	80	0.6	2,012.6	92	0.7	2,148.0	90	0.6	1,810.7	74	0.5	1,292.2	118	0.8	1,826.8	103	0.7	1,431.1	101	0.7	1,261.2	103	0.7	1,146.9	114	0.8	1,130.5	138	0.9	1,277.7	147	0.8	1,303.9	104	0.7	871.9	1,264	0.7	16,625.0
Rondônia	18	0.1	521.0	24	0.2	640.3	22	0.2	505.0	38	0.3	754.2	43	0.3	758.4	47	0.3	763.7	59	0.4	893.4	50	0.4	682.8	63	0.4	730.6	84	0.5	863.3	95	0.5	902.9	77	0.5	660.4	620	0.4	9,719.4
Roraima	16	0.1	1,300.8	17	0.1	1,328.1	16	0.1	1,136.8	15	0.1	884.4	19	0.1	816.0	31	0.2	734.3	54	0.4	428.1	130	0.9	317.3	211	1.5	238.9	236	1.5	193.9	365	2.1	253.2	369	2.5	204.9	1,479	0.9	17,570.5
Tocantins	7	0.1	1,308.4	3	0	515.9	3	0	430.1	7	0	838.3	9	0.1	943.4	12	0.1	1,125.2	11	0.1	915.5	7	0.1	520.8	3	0	204.2	15	0.1	970.6	14	0.1	872.0	5	0	296.4	96	0.1	8,465.6
Northeast	334	2.4	1,047.7	371	2.7	1,031.1	355	2.5	778.9	422	3.0	737.8	382	2.7	552.7	434	3.1	545.0	448	3.2	505.4	492	3.6	505.1	523	3.7	494.3	579	3.8	520.3	671	3.8	583.0	586	4.0	488.3	5,597	3.2	6,652.2
Alagoas	10	0.1	1,042.8	12	0.1	1,116.3	10	0.1	740.7	14	0.1	838.3	4	0	202.2	8	0.1	357.8	12	0.1	482.6	12	0.1	434.6	24	0.2	792.2	18	0.1	560.0	12	0.1	356.0	17	0.1	475.1	153	0.1	6,479.6
Bahia	137	1.0	1,107.5	166	1.2	1,219.6	152	1.1	930.5	191	1.3	990.1	151	1.1	686.7	164	1.2	671.1	184	1.3	684.6	204	1.5	696.1	202	1.4	640.5	216	1.4	654.3	250	1.4	732.9	209	1.4	588.9	2,226	1.3	8,675.9
Ceará	44	0.3	734.6	53	0.4	756.3	57	0.4	610.9	64	0.4	526.5	67	0.5	434.6	73	0.5	396.1	63	0.5	305.1	74	0.5	328.8	80	0.6	330.3	90	0.6	355.3	121	0.7	464.3	99	0.7	364.3	885	0.5	4,529.8
Maranhão	22	0.2	1,305.6	13	0.1	667.7	21	0.1	808.5	9	0.1	270.1	14	0.1	346.0	17	0.1	353.5	20	0.1	363.3	18	0.1	291.0	27	0.2	394.2	27	0.2	373.0	47	0.3	626.9	36	0.2	461.0	271	0.2	5,255.0
Paraíba	15	0.1	856.2	14	0.1	713.7	16	0.1	639.0	25	0.2	764.6	19	0.1	460.9	18	0.1	375.5	25	0.2	475.6	24	0.2	420.4	25	0.2	404.8	28	0.2	431.4	35	0.2	522.0	32	0.2	457.8	276	0.2	5,492.8
Pernambuco	53	0.4	1,027.9	64	0.5	1,115.0	63	0.4	871.5	70	0.5	746.9	69	0.5	585.7	83	0.6	600.9	94	0.7	608.1	100	0.7	583.2	99	0.7	527.1	118	0.8	596.8	137	0.8	671.6	110	0.8	518.7	1,060	0.6	7,242.9
Piauí	4	0	923.8	3	0	596.4	3	0	423.4	6	0	601.8	4	0	319.9	9	0.1	616.9	6	0	342.7	8	0.1	384.0	8	0.1	329.9	10	0.1	373.7	6	0	213.6	10	0.1	340.0	77	0	4,797.5
Rio Grande do Norte	31	0.2	1,090.4	36	0.3	1,083.4	19	0.1	427.0	29	0.2	504.8	41	0.3	595.2	48	0.3	615.8	39	0.3	454.1	37	0.3	396.3	43	0.3	428.1	58	0.4	547.1	47	0.3	424.1	54	0.4	465.0	482	0.3	5,884.1
Sergipe	18	0.1	2,608.7	10	0.1	1,232.3	14	0.1	1,302.3	14	0.1	1,023.0	13	0.1	788.8	14	0.1	749.5	5	0	241.7	15	0.1	631.4	15	0.1	547.0	14	0.1	474.6	16	0.1	522.9	19	0.1	594.6	167	0.1	8,484.7
Midwest	454	3.3	1,780.4	407	3.0	1,501.3	459	3.3	1,410.5	477	3.3	1,180.6	517	3.6	1,082.9	492	3.5	896.5	450	3.3	712.9	480	3.5	671.9	591	4.2	733.9	627	4.1	712.6	1,074	6.0	1,146.5	749	5.1	742.2	6,777	3.9	11,486.0
Federal District	114	0.8	1,720.2	97	0.7	1,355.2	92	0.7	969.3	129	0.9	995.1	134	0.9	857.6	95	0.7	506.7	101	0.7	432.2	115	0.8	426.2	127	0.9	435.5	166	1.1	541.2	170	1.0	534.3	98	0.7	294.4	1,438	0.8	6,829.0
Goiás	70	0.5	1,108.8	59	0.4	872.6	94	0.7	1,197.9	99	0.7	1,072.8	74	0.5	692.4	94	0.7	782.3	74	0.5	556.3	80	0.6	538.4	130	0.9	775.2	119	0.8	650.6	431	2.4	2,240.4	277	1.9	1,367.8	1,601	0.9	12,647.1
Mato Grosso	66	0.5	1,965.5	57	0.4	1,568.5	52	0.4	1,105.7	64	0.4	1,007.1	73	0.5	899.2	79	0.6	819.9	65	0.5	596.4	84	0.6	668.6	76	0.5	503.3	102	0.7	592.3	123	0.7	658.7	91	0.6	443.4	932	0.5	9,077.6
Mato Grosso do Sul	204	1.5	2,216.9	194	1.4	2,030.0	221	1.6	2,104.6	185	1.3	1,560.4	236	1.7	1,772.8	224	1.6	1,547.0	210	1.5	1,349.7	201	1.5	1,179.6	258	1.8	1,323.8	240	1.6	1,100.7	350	2.0	1,461.2	283	1.9	1,053.8	2,806	1.6	18,683.0
Southeast	11,329	82.5	3,287.5	11,244	82.6	3,053.4	11,580	82.1	2,749.1	11,677	81.9	2,431.9	11,618	81.5	2,172.6	11,460	80.6	1,966.8	11,207	81.0	1,795.0	10,935	79.0	1,647.9	10,926	76.9	1,550.0	11,647	76.0	1,592.4	13,094	73.6	1,754.9	10,740	73.5	1,396.4	137,457	79.1	22,776.3
Espírito Santo	66	0.5	1,433.5	72	0.5	1,433.0	60	0.4	932.0	60	0.4	719.6	56	0.4	573.5	52	0.4	484.9	56	0.4	487.5	62	0.4	510.0	54	0.4	419.5	65	0.4	484.9	90	0.5	653.1	72	0.5	505.8	765	0.4	6,888.5
Minas Gerais	218	1.6	1,142.6	317	2.3	1,537.1	318	2.3	1,299.7	323	2.3	1,100.5	351	2.5	1,023.4	329	2.3	845.5	324	2.3	748.8	345	2.5	723.5	355	2.5	672.7	370	2.4	653.1	468	2.6	792.5	362	2.5	581.9	4,080	2.3	9,929.4
Rio de Janeiro	3,120	22.7	3,598.7	3,148	23,1	3,398.1	3,157	22.4	2,985.9	3,194	22.4	2,658.4	3,266	22.9	2,447.5	3,177	22.3	2,194.3	3,102	22.4	2,024.1	2,975	21.5	1,867.4	3,108	21.9	1,886.4	3,246	21.2	1,937.7	3,518	19.8	2,086.2	2,764	18.9	1,611.0	37,775	21.7	25,349.2
São Paulo	7,925	57.7	3,383.5	7,707	56.6	3,083.4	8,045	57.1	2,826.9	8,100	56.8	2,513.1	7,945	55.7	2,224.0	7,902	55.5	2,035.3	7,725	55.8	1,855.5	7,553	54.5	1,699.5	7,409	52.1	1,561.5	7,966	52.0	1,613.1	9,018	50.7	1,786.9	7,542	51.6	1,447.4	94,837	54.6	23,574.1
South	1,405	10.2	1,954.0	1,362	10.0	1,757.3	1,483	10.5	1,580.7	1,450	10.2	1,226.0	1,445	10.1	996.5	1,533	10.8	906.0	1,388	10.0	727.8	1,543	11.1	713.2	1,599	11.3	636.1	1,705	11.1	607.8	1,966	11.1	646.9	1,724	11.8	513.3	18,603	10.7	10,337.1
Paraná	905	6.6	2,883.5	822	6.0	2,445.4	843	6.0	2,123.6	870	6.1	1,816.9	819	5.7	1,457.8	874	6.1	1,372.7	805	5.8	1,141.8	847	6.1	1,079.8	862	6.1	961.8	919	6.0	924.3	1,013	5.7	937.5	848	5.8	706.9	10,427	6.0	15,542.7
Rio Grande do Sul	328	2.4	1,236.5	355	2.6	1,249.1	464	3.3	1,374.1	381	2.7	914.1	406	2.8	804.7	437	3.1	746.7	358	2.6	544.8	398	2.9	532.5	452	3.2	521.6	471	3.1	492.7	537	3.0	528.8	505	3.5	462.3	5,092	2.9	8,197.6
Santa Catarina	172	1.3	1,229.2	185	1.4	1,195.9	176	1.2	864.8	199	1.4	693.3	220	1.5	573.4	222	1.6	472.2	225	1.6	412.7	298	2.2	471.7	285	2.0	379.4	315	2.1	368.4	416	2.3	441.1	371	2.5	347.9	3,084	1.8	6,075.4
Total	13,730	100	2,779.1	13,620	100	2,563.6	14,099	100	2,270.6	14,262	100	1,952.9	14,258	100	1,701.4	14,227	100	1,519.4	13,843	100	1,340.6	13,850	100	1,199.4	14,209	100	1,079.1	15,316	100	1,067.1	17,779	100	1,174.2	14,614	100	897.6	173,807	100	17,654.7

MC: mortality coefficient.

Regarding ICD-10 chapters, there was a change in the mortality profile due to the covid-19 pandemic (Table 4). In almost every year, most deaths resulted from “diseases of the circulatory system” (Chapter IX). However, in 2021 this profile shifted, with “certain infectious and parasitic diseases” (Chapter I) becoming the most frequent ICD-10 chapter. Over the study period, the chapters “neoplasms” (Chapter II), “diseases of the respiratory system” (Chapter X), and “external causes of morbidity and mortality” (Chapter XX) also ranked among the highest in absolute and relative frequencies (Table 4). In the analysis of the most frequent underlying causes of death during the study period, unspecified acute myocardial infarction (I21.9), unspecified pneumonia (J18.9), and coronavirus infection of unspecified site (B34.2) were the most common (Table 5).

**Table 4:** Mortality among international migrants residing in Brazil by ICD-10 chapter, 2011–2022.

Chapter number	ICD-10 – Chapter name	2011		2012		2013		2014		2015		2016		2017		2018		2019		2020		2021		2022		Total	
		n	%	n	%	n	%	n	%	n	%	n	%	n	%	n	%	n	%	n	%	n	%	n	%	n	%
I	Certain infectious and parasitic diseases	368	2.7	405	3.0	428	3.0	484	3.4	522	3.7	483	3.4	478	3.5	513	3.7	524	3.7	2,894	18.9	4,460	25.1	1,516	10.4	13,075	7.5
II	Neoplasms	2,619	19.1	2,614	19.2	2,669	18.9	2,645	18.5	2,684	18.8	2,700	19.0	2,605	18.8	2,656	19.2	2,572	18.1	2,371	15.5	2,544	14.3	2,434	16.7	31,113	17.9
III	Diseases of the blood and blood-forming organs and certain disorders involving the immune mechanism	85	0.6	92	0.7	85	0.6	100	0.7	83	0.6	101	0.7	78	0.6	99	0.7	92	0.6	101	0.7	99	0.6	104	0.7	1,119	0.6
IV	Endocrine, nutritional and metabolic diseases	809	5.9	747	5.5	757	5.4	766	5.4	752	5.3	699	4.9	702	5.1	676	4.9	747	5.3	722	4.7	788	4.4	680	4.7	8,845	5.1
V	Mental and behavioral disorders	198	1.4	164	1.2	198	1.4	215	1.5	177	1.2	166	1.2	153	1.1	196	1.4	193	1.4	168	1.1	192	1.1	191	1.3	2,211	1.3
VI	Diseases of the nervous system	584	4.3	648	4.8	712	5.1	747	5.2	771	5.4	784	5.5	763	5.5	746	5.4	789	5.6	702	4.6	760	4.3	770	5.3	8,776	5,0
VII	Diseases of the eye and adnexa	1	0	1	0	0	0	0	0	0	0	0	0	0	0	0	0	0	0	0	0	0	0	0	0	2	0
VIII	Diseases of the ear and mastoid process	0	0	2	0	2	0	0	0	0	0	1	0	1	0	2	0	2	0	0	0	0	0	2	0	12	0
IX	Diseases of the circulatory system	4,359	31.7	4,264	31.3	4,393	31,2	4,296	30.1	4,164	29.2	4,252	29.9	4,080	29.5	3,881	28.0	4,010	28.2	3,579	23.4	3,814	21.5	3,615	24.7	48,707	28.0
X	Diseases of the respiratory system	2,252	16.4	2,151	15.8	2,327	16.5	2,342	16.4	2,400	16.8	2,261	15.9	2.246	16,2	2,115	15.3	2,260	15.9	1,956	12.8	1,744	9.8	1,982	13.6	26,036	15.0
XI	Diseases of the digestive system	628	4.6	629	4.6	604	4.3	635	4.5	602	4.2	640	4.5	636	4.6	598	4.3	654	4.6	528	3.4	649	3.7	645	4.4	7,448	4.3
XII	Diseases of the skin and subcutaneous tissue	54	0.4	76	0.6	67	0.5	76	0.5	88	0.6	83	0.6	85	0.6	93	0.7	112	0.8	64	0.4	98	0.6	106	0.7	1,002	0.6
XIII	Diseases of the musculoskeletal system and connective tissue	73	0.5	81	0.6	73	0.5	93	0.7	90	0.6	76	0.5	92	0.7	85	0.6	93	0.7	67	0.4	86	0.5	72	0.5	981	0.6
XIV	Diseases of the genitourinary system	492	3.6	495	3.6	590	4.2	646	4.5	658	4.6	668	4.7	641	4.6	778	5.6	742	5.2	645	4.2	762	4.3	770	5.3	7,887	4.5
XV	Pregnancy, childbirth and the puerperium	6	0	4	0	0	0	13	0.1	7	0	14	0.1	10	0.1	22	0.2	18	0.1	26	0.2	34	0.2	22	0.2	176	0.1
XVI	Certain conditions originating in the perinatal period	4	0	4	0	1	0	3	0	1	0	1	0	4	0	2	0	1	0	3	0	14	0.1	19	0.1	57	0
XVII	Congenital malformations, deformations and chromosomal abnormalities	12	0.1	14	0.1	9	0.1	11	0.1	7	0	19	0.1	12	0.1	15	0.1	20	0.1	15	0.1	26	0.1	13	0.1	173	0.1
XVIII	Symptoms, signs and abnormal clinical and laboratory findings, not elsewhere classified	509	3.7	499	3.7	476	3.4	467	3.3	455	3.2	518	3.6	493	3.6	526	3.8	550	3.9	663	4.3	758	4.3	697	4.8	6,611	3.8
XX	External causes of morbidity and mortality	677	4.9	730	5.4	708	5.0	723	5.1	797	5.6	761	5.3	764	5.5	847	6.1	830	5.8	812	5.3	951	5.3	976	6.7	9,576	5.5
	Total	13,730	100	13,620	100	14,099	100	14,262	100	14,258	100	14,227	100	13,843	100	13,850	100	14,209	100	15,316	100	17,779	100	14,614	100	173,807	100

**Table 5:** Mortality among international migrants residing in Brazil by underlying cause of death, 2011–2022.

ICD-10	Underlying cause	2011		2012		2013		2014		2015		2016		2017		2018		2019		2020		2021		2022		Total	
		n	%	n	%	n	%	n	%	n	%	n	%	n	%	n	%	n	%	n	%	n	%	n	%	n	%
A419	Sepsis, unspecified organism	170	1.2	209	1.5	220	1.6	258	1.8	276	1.9	249	1.8	256	1.8	240	1.7	277	1.9	205	1.3	273	1.5	296	2.0	2,929	1.7
B342	Coronavirus infection, unspecified	0	0	0	0	0	0	0	0	0	0	0	0	0	0	0	0	0	0	2,431	15.9	3,842	21.6	904	6.2	7,177	4.1
C169	Malignant neoplasm of stomach, unspecified	194	1.4	188	1.4	200	1.4	209	1.5	170	1.2	183	1.3	163	1.2	175	1.3	152	1.1	128	0.8	129	0.7	104	0.7	1,995	1.1
C189	Malignant neoplasm of colon, unspecified	223	1.6	224	1.6	220	1.6	217	1.5	226	1.6	194	1.4	201	1.5	229	1.7	202	1.4	169	1.1	156	0.9	172	1.2	2,433	1.4
C20	Malignant neoplasm of rectum	77	0.6	79	0.6	95	0.7	97	0.7	93	0.7	98	0.7	91	0.7	79	0.6	58	0.4	58	0.4	69	0.4	82	0.6	976	0.6
C229	Malignant neoplasm of liver, not specified	60	0.4	53	0.4	66	0.5	64	0.4	58	0.4	70	0.5	81	0.6	58	0.4	77	0.5	64	0.4	70	0.4	58	0.4	779	0.4
C259	Malignant neoplasm of pancreas, unspecified	131	1.0	138	1.0	145	1.0	131	0.9	167	1.2	142	1.0	132	1.0	159	1.1	156	1.1	156	1.0	148	0.8	141	1.0	1,746	1.0
C349	Malignant neoplasm of unspecified part of bronchus or lung	326	2.4	316	2.3	351	2.5	323	2.3	332	2.3	352	2.5	329	2.4	360	2.6	311	2.2	293	1.9	299	1.7	271	1.9	3,863	2.2
C509	Malignant neoplasm of breast, unspecified	159	1.2	172	1.3	162	1.1	147	1.0	162	1.1	148	1.0	150	1.1	154	1.1	158	1.1	139	0.9	144	0.8	156	1.1	1,851	1.1
C61	Malignant neoplasm of prostate	227	1.7	256	1.9	233	1.7	230	1.6	204	1.4	230	1.6	238	1.7	194	1.4	217	1.5	194	1.3	201	1.1	183	1.3	2,607	1.5
C679	Malignant neoplasm of bladder, unspecified	90	0.7	79	0.6	89	0.6	97	0.7	106	0.7	86	0.6	99	0.7	107	0.8	103	0.7	93	0.6	105	0.6	91	0.6	1,145	0.7
C80	Malignant neoplasm without specification of site	72	0.5	62	0.5	80	0.6	83	0.6	82	0.6	73	0.5	64	0.5	62	0.4	57	0.4	58	0.4	59	0.3	61	0.4	813	0.5
E142	Unspecified diabetes mellitus - With renal complications	110	0.8	101	0.7	101	0.7	91	0.6	102	0.7	98	0.7	89	0.6	88	0.6	73	0.5	77	0.5	113	0.6	81	0.6	1,124	0.6
E149	Unspecified diabetes mellitus - Without complications	320	2.3	314	2.3	309	2.2	308	2.2	318	2.2	275	1.9	273	2.0	260	1.9	288	2.0	287	1.9	300	1.7	273	1.9	3,525	2.0
F03	Unspecified dementia	159	1.2	127	0.9	164	1.2	172	1.2	137	1.0	131	0.9	122	0.9	149	1.1	145	1.0	117	0.8	145	0.8	149	1.0	1,717	1.0
G20	Parkinson’s disease	76	0.6	99	0.7	93	0.7	108	0.8	112	0.8	116	0.8	102	0.7	117	0.8	106	0.7	99	0.6	112	0.6	109	0.7	1,249	0.7
G309	Alzheimer’s disease, unspecified	404	2.9	418	3.1	469	3.3	492	3.4	516	3.6	517	3.6	507	3.7	473	3.4	513	3.6	440	2.9	469	2.6	467	3.2	5,685	3.3
I10	Essential (primary) hypertension	197	1.4	197	1.4	211	1.5	231	1.6	221	1.6	202	1.4	207	1.5	198	1.4	222	1.6	294	1.9	304	1.7	242	1.7	2,726	1.6
I110	Hypertensive heart disease with heart failure	127	0.9	109	0.8	109	0.8	117	0.8	111	0.8	101	0.7	126	0.9	93	0.7	114	0.8	126	0.8	117	0.7	80	0.5	1,330	0.8
I219	Acute myocardial infarction, unspecified	1,072	7.8	1,072	7.9	1,074	7.6	1,100	7.7	1,129	7.9	1,035	7.3	1,030	7.4	951	6.9	1,026	7.2	931	6.1	900	5.1	821	5.6	12,141	7.0
I251	Atherosclerotic heart disease of native coronary artery	166	1.2	150	1.1	154	1.1	160	1.1	136	1.0	155	1.1	136	1.0	145	1.0	149	1.0	86	0.6	78	0.4	94	0.6	1,609	0.9
I255	Ischemic cardiomyopathy	116	0.8	113	0.8	106	0.8	99	0.7	100	0.7	93	0.7	96	0.7	93	0.7	118	0.8	66	0.4	74	0.4	61	0.4	1,135	0.7
I259	Chronic ischemic heart disease, unspecified	60	0.4	53	0.4	80	0.6	62	0.4	59	0.4	72	0.5	82	0.6	70	0.5	46	0.3	47	0.3	67	0.4	69	0.5	767	0.4
I269	Pulmonary embolism without acute cor pulmonale	110	0.8	99	0.7	110	0.8	113	0.8	118	0.8	131	0.9	114	0.8	102	0.7	89	0.6	78	0.5	104	0.6	121	0.8	1,289	0.7
I420	Dilated cardiomyopathy	110	0.8	114	0.8	131	0.9	89	0.6	73	0.5	98	0.7	79	0.6	70	0.5	68	0.5	37	0.2	39	0.2	58	0.4	966	0.6
I48	Atrial fibrillation and flutter	64	0.5	60	0.4	77	0.5	82	0.6	63	0.4	70	0.5	79	0.6	92	0.7	101	0.7	70	0.5	87	0.5	93	0.6	938	0.5
I499	Cardiac arrhythmia, unspecified	52	0.4	54	0.4	63	0.4	86	0.6	78	0.5	79	0.6	66	0.5	47	0.3	70	0.5	48	0.3	75	0.4	41	0.3	759	0.4
I500	Congestive heart failure	180	1.3	168	1.2	172	1.2	151	1.1	164	1.2	172	1.2	145	1.0	140	1.0	154	1.1	115	0.8	132	0.7	121	0.8	1,814	1.0
I509	Heart failure, unspecified	130	0.9	124	0.9	144	1.0	138	1.0	156	1.1	157	1.1	166	1.2	144	1.0	158	1.1	172	1.1	189	1.1	212	1.5	1,890	1.1
I619	Nontraumatic intracerebral hemorrhage, unspecified	154	1.1	173	1.3	175	1.2	184	1.3	145	1.0	172	1.2	137	1.0	152	1.1	152	1.1	141	0.9	157	0.9	150	1.0	1,892	1.1
I639	Cerebral infarction, unspecified	118	0.9	135	1.0	149	1.1	132	0.9	121	0.8	131	0.9	137	1.0	145	1.0	154	1.1	133	0.9	129	0.7	105	0.7	1,589	0.9
I64	Stroke, not specified as haemorrhage or infarction	418	3.0	362	2.7	349	2.5	327	2.3	334	2.3	317	2.2	281	2.0	242	1.7	237	1.7	230	1.5	258	1.5	227	1.6	3,582	2.1
I678	Other specified cerebrovascular diseases	139	1.0	142	1.0	126	0.9	140	1.0	105	0.7	134	0.9	100	0.7	93	0.7	90	0.6	83	0.5	89	0.5	84	0.6	1,325	0.8
I694	Sequelae of stroke, not specified as haemorrhage or infarction	193	1.4	189	1.4	176	1.2	139	1.0	156	1.1	173	1.2	127	0.9	121	0.9	103	0.7	95	0.6	93	0.5	95	0.7	1,660	1.0
J159	Unspecified bacterial pneumonia	94	0.7	64	0.5	73	0.5	92	0.6	89	0.6	113	0.8	132	1.0	169	1.2	197	1.4	186	1.2	238	1.3	237	1.6	1,684	1.0
J180	Bronchopneumonia, unspecified	489	3.6	489	3.6	499	3.5	491	3.4	515	3.6	393	2.8	397	2.9	377	2.7	360	2.5	181	1.2	206	1.2	293	2.0	4,690	2.7
J189	Pneumonia, unspecified	731	5.3	747	5.5	838	5.9	868	6.1	892	6.3	930	6.5	883	6.4	753	5.4	821	5.8	462	3.0	430	2.4	587	4.0	8,942	5.1
J440	Chronic obstructive pulmonary disease with acute lower respiratory infection	276	2.0	246	1.8	262	1.9	240	1.7	270	1.9	216	1.5	227	1.6	198	1.4	221	1.6	114	0.7	119	0.7	150	1.0	2,539	1.5
J449	Chronic obstructive pulmonary disease	187	1.4	173	1.3	144	1.0	146	1.0	166	1.2	156	1.1	138	1.0	135	1.0	154	1.1	111	0.7	98	0.6	130	0.9	1,738	1.0
J690	Pneumonitis due to inhalation of food and vomit	78	0.6	87	0.6	104	0.7	99	0.7	86	0.6	96	0.7	104	0.8	100	0.7	120	0.8	97	0.6	163	0.9	131	0.9	1,265	0.7
J988	Other specified respiratory disorders	28	0.2	21	0.2	29	0.2	35	0.2	20	0.1	17	0.1	18	0.1	12	0.1	18	0.1	434	2.8	110	0.6	16	0.1	758	0.4
K746	Other and unspecified cirrhosis of liver	57	0.4	59	0.4	62	0.4	51	0.4	69	0.5	57	0.4	53	0.4	61	0.4	57	0.4	46	0.3	59	0.3	65	0.4	696	0.4
N189	Chronic kidney disease, unspecified	62	0.5	59	0.4	65	0.5	73	0.5	72	0.5	68	0.5	80	0.6	77	0.6	72	0.5	67	0.4	77	0.4	75	0.5	847	0.5
N390	Urinary tract infection, site not specified	274	2.0	292	2.1	354	2.5	408	2.9	405	2.8	431	3.0	401	2.9	469	3.4	452	3.2	381	2.5	449	2.5	455	3.1	4,771	2.7
R54	Age-related physical debility	94	0.7	92	0.7	95	0.7	103	0.7	80	0.6	89	0.6	71	0.5	82	0.6	61	0.4	76	0.5	85	0.5	111	0.8	1,039	0.6
R98	Unattended death	78	0.6	72	0.5	62	0.4	56	0.4	48	0.3	63	0.4	55	0.4	49	0.4	56	0.4	64	0.4	54	0.3	51	0.3	708	0.4
R99	Ill-defined and unknown cause of mortality	230	1.7	228	1.7	205	1.5	194	1.4	218	1.5	250	1.8	249	1.8	295	2.1	322	2.3	400	2.6	481	2.7	407	2.8	3,479	2.0
W189	Striking against other object with subsequent fall	58	0.4	48	0.4	60	0.4	65	0.5	88	0.6	79	0.6	59	0.4	66	0.5	70	0.5	40	0.3	53	0.3	43	0.3	729	0.4
Y349	Unspecified event, undetermined intent	61	0.4	67	0.5	53	0.4	52	0.4	61	0.4	49	0.3	69	0.5	78	0.6	89	0.6	81	0.5	104	0.6	105	0.7	869	0.5
-	Others	4,729	34.4	4,727	34.7	4,791	34.0	4,912	34.4	4,849	34.0	4,966	34.9	4,902	35.4	5,127	37.0	5,195	36.6	4,816	31.4	5,527	31.1	5,486	37.5	60,027	34.5
-	Total	13,730	100	13,620	100	14,099	100	14,262	100	14,258	100	14,227	100	13,843	100	13,850	100	14,209	100	15,316	100	17,779	100	14,614	100	173,807	100

Finally, when analyzing deaths among individuals from countries that have faced or are facing migration crises and have vulnerable migrant populations residing in Brazil in recent years, we observed that, with the exception of Syria, unknown causes, preventable causes, and violence were among the 10 leading underlying causes of death for each country, namely: Angola (ill-defined causes and breast cancer), Haiti (ill-defined causes and HIV/Aids), Syria (same causes of death as those of international migrants from European countries), and Venezuela (ill-defined causes, cervical cancer, breast cancer, and violence by firearms or by sharp or penetrating objects). Covid-19 appeared among the 10 leading causes of death among migrants from all these countries of origin.

## DISCUSSION

This study used, for the first time, available secondary data to calculate MC among international migrants in Brazil. It also presented, for the first time, the overall mortality of this population group according to demographic characteristics, causes of death, and geographic distribution of these deaths. The data indicate a high number of deaths in 2021, reflecting the impact of the covid-19 pandemic, which significantly altered the mortality profile of international migrants residing in Brazil.

The predominance of deaths among men, older adults, White individuals, and those who were widowed or married is consistent with existing literature on overall mortality, suggesting vulnerability to noncommunicable chronic diseases and health conditions associated with aging^4,8,17^. The higher frequency of deaths among migrants from countries such as Portugal, Japan, and Italy can be explained by historical migration waves in the early 20^th^ century, resulting in an aging migrant population currently residing in Brazil^3,18^.

The highest MC among migrants born in Lithuania, Serbia, and Hungary, among others, may be attributed both to denominators with small populations, artificially inflating MC, and to pre-existing health conditions and unequal access to healthcare services^19^. The concentration of deaths in the Southeast and South, particularly in states of São Paulo, Rio de Janeiro, and Paraná, is consistent with the demographic distribution of migrants in the country, in regions more favorable for seeking work and income^22^.

The covid-19 pandemic had a significant impact on the mortality profile of migrants, with “diseases of the circulatory system” being surpassed by “certain infectious and parasitic diseases” as the most frequent cause of death in 2021, as was also observed in the general Brazilian population^23^. This shows that these populations are affected by public health emergency crises and highlights the need for specific health policies, particularly those prioritizing vaccination for vulnerable international migrant populations^6^.

The most frequent underlying causes of death align with the sociodemographic profile of these deaths, as they generally occur predominantly among older individuals^24^. A high prevalence of preventable causes of death and violence is also observed among international migrants born in countries that were or are currently experiencing economic crises and facing public health emergencies or humanitarian crises (outbreaks, epidemics, disasters, lack of assistance, climate change, conflicts, and wars)^4,6,25^. These data suggest that migrants from such regions face additional barriers in accessing healthcare and may be exposed to more precarious living conditions, contributing to higher frequencies of deaths from preventable causes.

A study on the mortality of internal and international migrants in Brazil, using data from the cohort of 100 million Brazilians of the *Centro de Integração de Dados e Conhecimentos para Saúde da Fundação Oswaldo Cruz* (Cidacs/Fiocruz – Center for Data and Knowledge Integration for Health of the Oswaldo Cruz Foundation), reached results and conclusions similar to those of this study, especially regarding the need for public health policies that effectively encompass different socioeconomic characteristics and specificities of international migrants residing in Brazil^17^.

Although Brazil has made progress in terms of public policies for international migrants —such as the Migration Law of 2017, Brazil’s rejoining of the Global Compact for Safe, Orderly and Regular Migration, and the creation of the Working Group for the establishment of the National Health Policy for Migrant, Refugee and Stateless Populations—significant challenges remain^28^. The invisibility of international migrants in health statistics, due to the incomplete data and the lack of mandatory completion of important variables in health surveillance systems, hinders the implementation of effective public health strategies.

This study presents limitations. The 2022 IBGE Census results regarding migrant populations were not released until the end of this research, making it impossible to produce more precise estimates of denominators for calculating MC. The denominators did not consider, for example, undocumented international migrants in Brazil or international migrants who emigrated during the study period. It was not possible to compare MC of international migrants residing in Brazil with those of Brazilians residing in Brazil, as we believe these populations have different age structures (each country of birth has a distinct age structure) and differ from the Brazilian population. This prevents the calculation of standardized MC, which would enable such a comparison.

It was not possible to perform linear interpolation for migrant populations by municipality due to frequent changes of residence between nearby cities, which made analysis at the mesoregional level unfeasible. We sought to minimize this limitation by conducting the analysis at the macroregion and FU levels, as these are larger units of analysis that reduce the potential for bias.

Despite its limitations, this study reinforces the urgent need to include international migrants in public health policies, recognizing them as a vulnerable and priority population. Specific policies that ensure equity in access to healthcare services, medications, and vaccines are essential to improve the health conditions of this population in the long term. It is crucial to enhance data collection and the integration of migrants into health information systems to enable better preparedness, surveillance, and response to their health needs.

## Footnotes

a The populations calculated from 2011 to 2022 used as denominators can be accessed at: https://github.com/joaorobertobr/denominadores_migrantes.

## References

[1] Nolasco C (2016). Migrações internacionais: Conceitos, tipologia e teorias.

[2] International Organization for Migration (2024). World Migration Report 2024.

[3] Levy MSF (1974). O papel da migração internacional na evolução da população brasileira (1872 a 1972).. Rev Saúde Pública.

[4] Ventura DFL, Yujra VQ (2019). Saúde de migrantes e refugiados.

[5] Cavalcante JR, Proença R, Cano I, Trajman A, Faerstein E (2022). Perfil sociodemográfico e de saúde de solicitantes de refúgio no Rio de Janeiro, 2016-2017. Rev Saúde Pública.

[6] Rodrigues IA, Cavalcante JR, Faerstein E (2020). Pandemia de Covid-19 e a saúde dos refugiados no Brasil. Physis.

[7] Ministério da Saúde (BR) (2024). Sistema de Informação sobre Mortalidade (SIM) - Estrutura.

[8] National Research Council (US) (2001). Roundtable on the Demography of Forced Migration. Forced Migration & Mortality [Internet]..

[9] Instituto Brasileiro de Geografia e Estatística (2024). Canais IBGE [Internet]. Brasília: IBGE.

[10] Wells RHC, Bay-Nielsen H, Braun R, Israel RA, Laurenti R, Maguin P (2011). CID-10: classificação estatística internacional de doenças e problemas relacionados à saúde.

[11] Instituto Brasileiro de Geografia e Estatística (2010). Censo 2010 [Internet]..

[12] Ministério da Justiça e Segurança Pública (BR) (2023). Microdados - Portal de Imigração [Internet]..

[13] Tavares R, Moraes CL (1994). Comparações de coeficientes gerais de mortalidade.

[14] Junger G, Cavalcanti L, Oliveira T, Lemos SF, Tonhati T, Costa LFL (2023). Refúgio em Números 2023.

[15] Brazil (2018). Lei n° 13.709, de 14 de agosto de 2018. Diário Oficial da União.

[16] Ministério da Saúde (BR) Resolução n° 510, de 7 de abril de 2016. Brasília: Ministério da Saúde; 2016.

[17] Pescarini JM, Goes EF, Pinto PFPS, Dos Santos BPS, Machado DB, Abubakar I (2023). Mortality among over 6 million internal and international migrants in Brazil: a study using the 100 Million Brazilian Cohort. The Lancet Regional Health - Americas.

[18] Freitas SMD (1999). E chegam os imigrantes... O café e a imigração em São Paulo.

[19] Santos HS, Medeiros AA (2017). Migração e acesso aos serviços de saúde: a necessidade da pauta intercultural para o cumprimento dos direitos humanos.

[20] Miyashiro C (2018). Acesso aos serviços de saúde pelas populações migrantes: revisão sistemática.

[21] Aragão HT, Menezes AN, Oliveira ML L, Santana JT, Madi RR, Melo CM de (2023). Demandas e utilização de serviços de saúde entre imigrantes de uma região metropolitana do nordeste do Brasil. Esc Anna Nery.

[22] Cavalcanti L, Oliveira T, Silva SFL (2023). Relatório Anual OBMigra 10 anos: pesquisas, dados e contribuições para políticas públicas.

[23] Ministério da Saúde (BR) (2024). Painel Coronavírus.

[24] Ministério da Saúde (BR) (2022). Boletim Epidemiológico 02: Mortalidade de idosos no Brasil em 2000, 2009 e 2019.

[25] Proença R, Cavalcante JR, Trajman A, Faerstein E (2023). Violências relatadas por solicitantes de refúgio atendidos na Cáritas Arquidiocesana do Rio de Janeiro de 2010 a 2017. Rev Bras Estud Popul.

[26] Organização das Nações Unidas (2021). Mudanças climáticas impulsionam migrações e deslocamentos forçados.

[27] Pozzi GS (2024). Crise climática: impacto dos desastres na migração forçada.

[28] Brazil (2017). Lei n° 13.445, de 24 de maio de 2017. Diário Oficial da União.

[29] Ministério das Relações Exteriores (BR) (2023). Retorno do Brasil ao Pacto Global para Migração Segura, Ordenada e Regular.

[30] Ministério da Saúde (BR), Ministério da Saúde cria Grupo de Trabalho para elaborar a Política Nacional de Saúde de refugiados (2023). https://www.gov.br/saude/pt-br/assuntos/noticias/2023/junho/ministerio-da-saude-cria-grupo-de-trabalho-para-elaborar-a-politica-nacional-de-saude-de-refugiados.

