# Supplementary material for: Profile of deaths and causes of mortality among international migrants in Brazil, 2011–2022
Source: Rev Saude Publica. 2026 Jan 12;59:e46. doi: 10.11606/s1518-8787.2025059006519 (PMC12713528; doi:10.11606/s1518-8787.2025059006519)
Supplement: Translation

# Translation: Perfil de óbitos e causas da mortalidade de migrantes internacionais no Brasil, 2011–2022

**Authors:** João Roberto Cavalcante, Anete Trajman, Eduardo Faerstein

**Affiliations:** I Universidade do Estado do Rio de Janeiro. Instituto de Medicina Social Hesio Cordeiro. Rio de Janeiro, RJ, Brasil; II Universidade Federal do Rio de Janeiro. Departamento de Clínica Médica. Rio de Janeiro, RJ, Brasil; III McGill University. McGill International TB Centre. Montreal, Canada; IV Rede Brasileira de Pesquisa em Tuberculose. Rio de Janeiro, RJ, Brasil

**Keywords:** Migrantes, Mortalidade, Epidemiologia, Saúde Global, Brasil

## Abstract

**OBJETIVO:**

Descrever o perfil de óbitos e analisar as causas da mortalidade em migrantes internacionais residentes no Brasil entre 2011 e 2022.

**MÉTODOS:**

Estudos transversal descritivo e ecológico com dados secundários. Foi analisado o perfil sociodemográfico dos óbitos ocorridos entre 2011 e 2022, e apresentadas as frequências absolutas, relativas e o coeficiente de mortalidade por 100 mil habitantes por país de nascimento, macrorregião e unidade federada de residência. Também foram analisadas as causas da mortalidade.

**RESULTADOS:**

Registraram-se 173.807 óbitos de migrantes internacionais entre 2011 e 2022, a maioria em 2021 (17.779; 10,2%). O perfil de óbitos predominante foi de homens (97.053; 55,8%), faixa-etária ≥ 81 anos (104.308; 60,0%), raça/cor da pele branca (136.835; 78,7%) e viúvos (72.156; 41,5%). A maioria dos migrantes falecidos nasceram em Portugal (64.909; 37,3%), Japão (22.748; 13,1%), Itália (16.178; 9,3%) e Espanha (12.835; 7,3%). Os maiores coeficientes de mortalidade foram de migrantes nascidos na Lituânia (190.079/100.000), Sérvia (146.794/100.000) e Hungria (96.395/100.000). As macrorregiões Sudeste (137.457; 79,1%) e Sul (18.603; 10,7%) do Brasil concentraram a maioria dos óbitos, com maiores coeficientes de mortalidade nos estados do Rio de Janeiro (25.349/100.000), São Paulo (23.574/100.000) e Mato Grosso do Sul (18.683/100.000). A pandemia de covid-19 aumentou os óbitos por doenças infecciosas e parasitárias a partir de 2020, tendo pico em 2021 (4.460; 25,0% dos óbitos do ano). A causa básica mais frequente no período foi infarto agudo do miocárdio não especificado (12.141; 7,0%).

**CONCLUSÃO:**

Este estudo evidenciou a necessidade de ações de saúde direcionadas para os perfis de migrantes internacionais com maior risco de morrer e por causas de óbitos; porém, a invisibilidade dessa população nos indicadores de saúde pública, de forma geral, dificulta a implementação de estratégias eficazes. Políticas que garantam acesso equitativo a serviços de saúde, medicamentos e vacinas são essenciais para melhorar a saúde dessa população a longo prazo.

## INTRODUÇÃO

A migração internacional pode ser definida como o deslocamento de pessoas de seus países de origem para residir temporária ou permanentemente em outro país^1^. Esse movimento pode ser desencadeado por fatores econômicos, sociais e políticos^1^. A integração de migrantes internacionais nos países de destino é complexa em diversos aspectos, principalmente no campo dos direitos humanos e das políticas públicas^1^.

Há tendência crescente desse fenômeno nos últimos anos tanto no Brasil quanto em outros países. Os registros mais recentes apontam que havia 281 milhões de migrantes internacionais no mundo até 2020, sendo 1,6 milhão no Brasil^2^. O país tem recebido significativos fluxos migratórios ao longo da sua história, principalmente de migrantes internacionais vindos de Portugal, Japão, Itália e Alemanha, além de pessoas escravizadas e trazidas contra sua vontade de países africanos como Nigéria, Costa do Marfim, Congo, Angola e Moçambique^3^. Nos últimos 15 anos, o Brasil tem experienciado um aumento considerável na migração de populações em situação de vulnerabilidade provenientes de países da América do Sul e Central, destacando-se os migrantes venezuelanos e haitianos^2^.

No âmbito da saúde, os migrantes internacionais encontram-se frequentemente em situação de vulnerabilidade^4^. A depender do país de origem, as condições de saúde desse grupo são preocupantes, pois frequentemente são vítimas de violência e/ou expostos a doenças infecciosas antes e durante a migração internacional^4^. Essas populações mais vulnerabilizadas podem apresentar doenças e agravos não tratados, como sofrimentos mental e psicossocial, além de doenças crônicas não transmissíveis^4,5^. A barreira do idioma, a falta de conhecimento sobre os sistemas de saúde locais e o estresse do processo migratório contribuem para tal cenário^4^.

No Brasil, a carência de dados específicos sobre a saúde dessas populações gera dificuldades para a formulação de políticas públicas eficazes^6^. O Sistema de Informações sobre Mortalidade (SIM) potencialmente representa ferramenta essencial para suprir essa falta de informações, uma vez que inclui o registro do país de nascimento dos indivíduos falecidos^7^. Entretanto, apesar dos dados do SIM estarem disponíveis desde 1979, até o momento não se tem conhecimento de análises e divulgação dessas informações em nível nacional, possivelmente em razão da necessidade de serem calculados denominadores para os coeficientes de mortalidade (CM), que possibilitariam as comparações^8^.

Analisar e descrever essas causas, para a população de migrantes internacionais residentes no Brasil, é essencial para o desenvolvimento de estratégias de gestão em saúde que possam diminuir o risco de óbitos por causas evitáveis e aumentar o acesso aos serviços de saúde por essa população. O objetivo deste estudo foi descrever o perfil de óbitos e analisar as causas da mortalidade em migrantes internacionais residentes no Brasil entre 2011 e 2022.

## MÉTODOS

Trata-se de um estudo transversal descritivo e ecológico com dados secundários. As unidades de análise foram os países de nascimento, as unidades federadas (UF), as macrorregiões de residência no Brasil e as causas básicas de óbito.

O Brasil é composto por 5.570 municípios, organizados em 26 estados e um Distrito Federal, formando cinco grandes macrorregiões^9^. Com uma área de 8.515.767 km^2^, é o quinto maior país do mundo em termos de extensão territorial^9^. Em 2022, o país tinha uma população estimada de 203 milhões de habitantes, sendo o sétimo país mais populoso do mundo, com uma densidade demográfica de aproximadamente 25,2 habitantes por km^2 9^.

Foram incluídos todos os óbitos registrados de migrantes internacionais no período de 2011 a 2022. O ano de 2023 não entrou na pesquisa porque os dados disponíveis estavam incompletos no momento de obtenção dos bancos. Para separação de migrantes internacionais de brasileiros natos nos bancos do SIM, foi utilizada a variável “NATURAL”, que possui boa completude, uma vez que a informação sobre naturalidade é obrigatória na declaração de óbito^7^. Essa variável, registrada no banco de dados do SIM, é de acesso aberto no sítio do “OpenDATASUS” do Ministério da Saúde do Brasil^7^.

Analisaram-se as frequências absolutas e relativas do ano do óbito, sexo, faixa etária, raça/cor da pele, estado civil, capítulo da CID-10 e causa básica do óbito^10^. Para o cálculo dos CM por 100 mil habitantes, foi necessária a elaboração de denominadores da população de migrantes residentes no Brasil, por país de nascimento, UF e macrorregiões no período do estudo. Foram utilizados os dados abertos do Censo Demográfico de 2010 do Instituto Brasileiro de Geografia e Estatística e do Sistema de Registro Nacional Migratório (Sismigra) do Ministério da Justiça (2011 a 2022), que reúne os números de migrantes internacionais com registro ativo no país, além dos dados de óbitos do SIM (2011 a 2022)^7,11,12^. A completude das variáveis desses sistemas são boas, posto vez que a variável que identifica se o indivíduo é migrante internacional é de preenchimento obrigatório^7,11,12^. Todas as categorias do Sismigra (residente, temporário, provisório e fronteiriço) foram consideradas^12^. Utilizando esses dados foi possível estimar pela primeira vez as populações de migrantes internacionais residentes no Brasil de 2011 a 2022. A fórmula para o cálculo dessas populações para o ano de 2011 foi:


Y=A+B−C

Y = população estimada de migrantes internacionais residentes no Brasil por país de nascimento, macrorregião ou UF (2011);

A = número de migrantes internacionais residentes no Brasil por país de nascimento, macrorregião ou UF em 2010 (Censo 2010);

B = número de migrantes internacionais que entraram no Brasil por país de nascimento, macrorregião ou UF em 2011 (Sismigra, 2011);

C = número de óbitos de migrantes internacionais no Brasil por país de nascimento, macrorregião ou UF em 2011 (SIM, 2011).

Para as populações de 2012 a 2022 não foi necessário mais o uso do Censo de 2010 de forma separada, pois este já estava incorporado na população calculada para o ano de 2011. A fórmula utilizada para o cálculo das populações dos anos seguintes foi:


Y=A+B−C

Y = população estimada de migrantes internacionais residentes no Brasil por país de nascimento, macrorregião ou UF no ano vigente;

A = número de migrantes internacionais residentes no Brasil por país de nascimento, macrorregião ou UF do ano anterior (calculado pelos autores);

B = número de migrantes internacionais que entraram no Brasil por país de nascimento, macrorregião ou UF no ano vigente (Sismigra);

C = número de óbitos de migrantes internacionais no Brasil por país de nascimento, macrorregião ou UF no ano vigente (SIM).

Foi utilizada a população do meio do período no denominador dos CM^a^, sendo considerada a data de 2 de julho como o meio do período de cada ano^13^, conforme demonstrado na fórmula descrita abaixo:


$$CM=\frac{\text{ Número de óbitos }}{\text{ População do meio do período }}\times 100.000$$

Durante a análise, constatou-se a necessidade de explorar as principais causas de óbitos de migrantes vindos de alguns países específicos que tenham enfrentado ou estejam enfrentando crise migratória, ou que tenham populações de migrantes vulnerabilizados residindo no Brasil nos últimos anos, a saber: Angola, Haiti, Síria e Venezuela^14^.

O *software* RStudio 3.3.0+ foi operado para processamento e análise de dados. Por se tratar de um estudo com dados secundários, agregados e de acesso livre, não houve necessidade de aprovação por um comitê de ética em pesquisa, conforme a Resolução nº 510 de 2016 do Conselho Nacional de Ética em Pesquisa. Os bancos analisados eram públicos, anonimizados, segundo predito na Lei Geral de Proteção de Dados Pessoais (Lei n° 13.709/2018)^15,16^.

## RESULTADOS

Entre 2011 e 2022, registraram-se 173.807 óbitos de migrantes internacionais residentes no Brasil, sendo a maioria no ano de 2021 (Tabela 1). A maioria dos óbitos ocorreram em indivíduos do sexo masculino, idosos, com a média de idade de 79,8 anos. A raça/cor da pele mais predominante foi a branca, seguida da amarela; porém, no ano de 2021, a raça/cor da pele parda superou a amarela. A maior frequência de óbitos foi em indivíduos viúvos, seguidos de casados (Tabela 1).

**Tabela 1:** Perfil sociodemográfico dos óbitos de migrantes internacionais residentes no Brasil, 2011 a 2022.

Características sociodemográficas	2011		2012		2013		2014		2015		2016		2017		2018		2019		2020		2021		2022		Total	
	n	%	n	%	n	%	n	%	n	%	n	%	n	%	n	%	n	%	n	%	n	%	n	%	n	%
Ano do óbito	13.730	7,9	13.620	7,8	14.099	8,1	14.262	8,2	14.258	8,2	14.227	8,2	13.843	8,0	13.850	8,0	14.209	8,2	15.316	8,8	17.779	10,2	14.614	8,4	173.807	100
Sexo																										
Masculino	7.636	55,6	7.580	55,7	7.904	56,1	7.871	55,2	7.770	54,5	7.887	55,4	7.660	55,3	7.608	54,9	7.921	55,7	8.823	57,6	10.349	58,2	8.044	55,0	97.053	55,8
Feminino	6.094	44,4	6.039	44,3	6.194	43,9	6.391	44,8	6.488	45,5	6.339	44,6	6.183	44,7	6.241	45,1	6.288	44,3	6.488	42,4	7.427	41,8	6.563	44,9	76.735	44,1
Ignorado	0	0	1	0	1	0	0	0	0	0	1	0	0	0	1	0	0	0	5	0	3	0	7	0	19	0
Faixa etária (anos)																										
< 1	12	0,1	11	0,1	7	0	9	0,1	5	0	10	0,1	10	0,1	14	0,1	23	0,2	13	0,1	39	0,2	19	0,1	172	0,1
1 a 10	29	0,2	24	0,2	26	0,2	28	0,2	22	0,2	21	0,1	23	0,2	35	0,3	57	0,4	36	0,2	50	0,3	59	0,4	410	0,2
11 a 20	53	0,4	52	0,4	53	0,4	40	0,3	42	0,3	45	0,3	40	0,3	68	0,5	70	0,5	89	0,6	95	0,5	125	0,9	772	0,4
21 a 30	80	0,6	96	0,7	106	0,8	121	0,8	122	0,9	138	1,0	156	1,1	197	1,4	189	1,3	257	1,7	339	1,9	290	2,0	2.091	1,2
31 a 40	114	0,8	102	0,7	100	0,7	133	0,9	164	1,2	164	1,2	172	1,2	212	1,5	239	1,6	347	2,3	437	2,5	367	2,5	2.551	1,5
41 a 50	174	1,3	185	1,4	182	1,3	220	1,5	209	1,5	229	1,6	201	1,5	235	1,7	292	2,0	377	2,5	637	3,6	407	2,8	3.348	1,9
51 a 60	582	4,2	498	3,7	510	3,6	473	3,3	461	3,2	462	3,2	432	3,1	470	3,4	465	3,1	582	3,8	899	5,1	599	4,1	6.433	3,7
61 a 70	1.409	10,3	1.417	10,4	1.490	10,6	1.503	10,5	1.453	10,2	1.487	10,5	1.388	10,0	1.357	9,8	1.375	9,3	1.589	10,4	1.941	10,9	1.323	9,1	17.732	10,2
71 a 80	3.211	23,4	3.096	22,7	3.036	21,5	3.025	21,2	2.862	20,1	2.796	19,7	2.710	19,6	2.651	19,1	2.746	18,5	3.189	20,8	3.801	21,4	2.795	19,1	35.918	20,7
≥ 81	8.063	58,7	8.135	59,7	8.588	60,9	8.707	61,1	8.916	62,5	8.873	62,4	8.708	62,9	8.607	62,1	8.745	58,9	8.833	57,7	9.537	53,6	8.596	58,8	104.308	60,0
Ignorado	3	0	4	0	1	0	3	0	2	0	2	0	3	0	4	0	8	0,1	4	0	4	0	34	0,2	72	0
Raça/cor da pele																										
Branca	11.129	81,1	10.908	80,1	11.388	80,8	11.520	80,8	11.479	80,5	11.332	79,7	11.116	80,3	10.976	79,2	11.160	78,5	11.604	75,8	13.274	74,7	10.949	74,9	136.835	78,7
Preta	95	0,7	96	0,7	117	0,8	164	1,1	183	1,3	200	1,4	201	1,5	265	1,9	269	1,9	428	2,8	484	2,7	380	2,6	2.882	1,7
Amarela	1.674	12,2	1.721	12,6	1.736	12,3	1.743	12,2	1.740	12,2	1.812	12,7	1.668	12,0	1.565	11,3	1.494	10,5	1.656	10,8	1.776	10,0	1.519	10,4	20.104	11,6
Parda	424	3,1	463	3,4	477	3,4	515	3,6	626	4,4	638	4,5	630	4,6	780	5,6	992	7,0	1.348	8,8	1.912	10,8	1.491	10,2	10.296	5,9
Indígena	14	0,1	15	0,1	6	0	19	0,1	8	0,1	19	0,1	30	0,2	36	0,3	53	0,4	62	0,4	54	0,3	70	0,5	386	0,2
Ignorado	394	2,9	417	3,1	375	2,7	301	2,1	222	1,6	226	1,6	198	1,4	228	1,6	241	1,7	218	1,4	279	1,6	205	1,4	3.304	1,9
Estado civil																										
Solteiro(a)	1.323	9,6	1.278	9,4	1.378	9,8	1.417	9,9	1.407	9,9	1.441	10,1	1.404	10,1	1.554	11,2	1.705	12,0	1.998	13,0	2.465	13,9	2.173	14,9	19.543	11,2
Casado(a)	5.371	39,1	5.269	38,7	5.361	38,0	5.381	37,7	5.369	37,7	5.355	37,6	5.129	37,1	4.924	35,6	5.047	35,5	5.567	36,3	6.598	37,1	4.825	33,0	64.196	36,9
Viúvo(a)	5.887	42,9	5.836	42,8	6.069	43,0	6.198	43,5	6.201	43,5	6.056	42,6	5.970	43,1	5.908	42,7	5.895	41,5	5.867	38,3	6.457	36,3	5.812	39,8	72.156	41,5
Separado(a)/divorciado(a)	610	4,4	655	4,8	657	4,7	679	4,8	673	4,7	745	5,2	764	5,5	812	5,9	840	5,9	952	6,2	1.074	6,0	906	6,2	9.367	5,4
União estável	107	0,8	161	1,2	139	1,0	159	1,1	169	1,2	198	1,4	198	1,4	248	1,8	251	1,8	348	2,3	403	2,3	276	1,9	2.657	1,5
Ignorado	432	3,1	421	3,1	495	3,5	428	3,0	439	3,1	432	3,0	378	2,7	404	2,9	471	3,3	584	3,8	782	4,4	622	4,3	5.888	3,4

A maior parte dos migrantes internacionais que evoluíram para óbitos eram de Portugal, seguidos de Japão, Itália, Espanha, Alemanha, Argentina, Bolívia, Paraguai, Líbano e Uruguai (Tabela 2). Os maiores CM no período foram de migrantes internacionais nascidos na Lituânia, seguidos de Sérvia, Hungria, Polônia, Jordânia, Japão, Portugal, Romênia, Egito e Líbano (Tabela 2).

**Tabela 2:** Mortalidade de migrantes internacionais residentes no Brasil por país de nascimento, 2011 a 2022.

País de nascimento	2011		2012		2013		2014		2015		2016		2017		2018		2019		2020		2021		2022		Período completo (2011 a 2022)	
	n (%)	CM	n (%)	CM	n (%)	CM	n (%)	CM	n (%)	CM	n (%)	CM	n (%)	CM	n (%)	CM	n (%)	CM	n (%)	CM	n (%)	CM	n (%)	CM	n (%)	CM
África do Sul	10 (0,1)	712,8	8 (0,1)	530,9	10 (0,1)	571,8	9 (0,1)	451,6	15 (0,1)	682,6	11 (0,1)	465,0	8 (0,1)	322,5	11 (0,1)	428,1	15 (0,1)	567,9	11 (0,1)	408,5	13 (0,1)	473,2	9 (0,1)	320,1	130 (0,1)	5.364,7
Alemanha	403 (2,9)	3.014,2	393 (2,9)	2.732,7	432 (3,1)	2.608,1	414 (2,9)	2.184,0	423 (3,0)	1.991,5	379 (2,7)	1.630,7	385 (2,8)	1.548,2	401 (2,9)	1.538,3	433 (3,0)	1.602,5	336 (2,2)	1.221,6	702 (3,9)	2.552,2	576 (3,9)	2.080,7	5.277 (3,0)	21.937,7
Angola	42 (0,3)	683,9	44 (0,3)	664,8	38 (0,3)	496,1	45 (0,3)	503,6	68 (0,5)	640,1	58 (0,4)	465,2	71 (0,5)	513,1	72 (0,5)	489,6	67 (0,5)	433,9	62 (0,4)	388,4	92 (0,5)	546,5	61 (0,4)	328,0	720 (0,4)	5.474,1
Argentina	250 (1,8)	981,9	292 (2,1)	1.061,4	300 (2,1)	933,2	291 (2,0)	770,6	280 (2,0)	648,0	318 (2,2)	663,9	342 (2,5)	656,1	360 (2,6)	640,8	364 (2,6)	598,6	362 (2,4)	560,7	475 (2,7)	705,5	392 (2,7)	549,3	4.026 (2,3)	8.050,4
Austrália	22 (0,2)	2.973,0	20 (0,1)	2.372,5	7 (0)	663,2	11 (0,1)	846,2	9 (0,1)	578,0	11 (0,1)	608,9	7 (0,1)	349,5	1 (0)	46,9	8 (0,1)	356,3	11 (0,1)	472,3	3 (0)	125,8	2 (0)	81,5	112 (0,1)	5.880,0
Áustria	71 (0,5)	4.159,3	64 (0,5)	3.619,9	64 (0,5)	3.359,6	83 (0,6)	4.037,0	52 (0,4)	2.335,5	63 (0,4)	2.624,5	64 (0,5)	2.529,1	63 (0,5)	2.407,3	64 (0,5)	2.391,2	64 (0,4)	2.378,7	62 (0,3)	2.299,3	52 (0,4)	1.893,7	766 (0,4)	31.068,7
Bélgica	22 (0,2)	1.406,6	26 (0,2)	1.496,4	27 (0,2)	1.279,3	34 (0,2)	1.347,9	31 (0,2)	1.060,2	29 (0,2)	888,8	26 (0,2)	731,6	36 (0,3)	945,9	44 (0,3)	1.095,9	28 (0,2)	675,8	55 (0,3)	1.308,9	41 (0,3)	956,3	399 (0,2)	11.706,0
Bolívia	197 (1,4)	464,7	189 (1,4)	394,0	218 (1,5)	367,4	242 (1,7)	352,8	262 (1,8)	349,3	273 (1,9)	339,2	225 (1,6)	261,3	293 (2,1)	313,6	328 (2,3)	325,1	504 (3,3)	477,2	657 (3,7)	598,5	453 (3,1)	392,5	3.841 (2,2)	4.611,1
Cabo Verde	27 (0,2)	2.124,3	5 (0)	359,2	11 (0,1)	664,5	7 (0)	353,1	7 (0)	302,6	1 (0)	39,1	8 (0,1)	292,0	10 (0,1)	347,3	7 (0)	231,9	9 (0,1)	288,4	10 (0,1)	317,0	10 (0,1)	312,6	112 (0,1)	4.227,6
Chile	113 (0,8)	827,2	127 (0,9)	890,2	134 (1,0)	864,0	158 (1,1)	938,3	142 (1,0)	782,9	154 (1,1)	797,7	175 (1,3)	859,4	185 (1,3)	868,2	187 (1,3)	841,3	263 (1,7)	1.149,2	327 (1,8)	1.403,0	233 (1,6)	977,1	2.198 (1,3)	11.081,7
China	148 (1,1)	819,1	167 (1,2)	832,6	157 (1,1)	643,0	174 (1,2)	590,8	163 (1,1)	474,4	187 (1,3)	485,1	174 (1,3)	409,6	200 (1,4)	432,9	186 (1,3)	377,7	223 (1,5)	439,5	351 (2,0)	685,7	228 (1,6)	439,5	2.358 (1,4)	5.820,5
Colômbia	20 (0,1)	262,2	33 (0,2)	349,0	26 (0,2)	176,1	38 (0,3)	174,5	50 (0,4)	172,5	57 (0,4)	155,8	48 (0,3)	106,6	53 (0,4)	97,4	72 (0,5)	113,3	103 (0,7)	148,4	157 (0,9)	214,5	102 (0,7)	131,4	759 (0,4)	1.859,9
Coreia do Sul	83 (0,6)	976,1	76 (0,6)	820,4	80 (0,6)	744,1	75 (0,5)	602,7	77 (0,5)	525,6	97 (0,7)	584,7	107 (0,8)	605,7	90 (0,6)	489,4	117 (0,8)	613,3	118 (0,8)	602,5	139 (0,8)	695,5	107 (0,7)	524,8	1.166 (0,7)	6.808,0
Cuba	11 (0,1)	627,9	12 (0,1)	634,8	12 (0,1)	246,1	16 (0,1)	141,9	22 (0,2)	138,4	28 (0,2)	145,6	26 (0,2)	107,2	22 (0,2)	78,5	24 (0,2)	80,2	46 (0,3)	148,2	59 (0,3)	185,9	35 (0,2)	103,8	313 (0,2)	1.439,8
Egito	75 (0,5)	6.097,6	80 (0,6)	6.184,8	77 (0,5)	5.565,6	69 (0,5)	4.760,3	97 (0,7)	6.419,6	76 (0,5)	4.772,4	88 (0,6)	5.114,8	75 (0,5)	4.088,3	67 (0,5)	3.471,5	80 (0,5)	4.006,0	121 (0,7)	6.025,9	87 (0,6)	4.288,9	992 (0,6)	59.885,3
Equador	16 (0,1)	1.009,5	13 (0,1)	720,6	5 (0)	215,5	9 (0,1)	298,1	8 (0,1)	204,7	5 (0)	100,3	7 (0,1)	115,3	11 (0,1)	156,3	7 (0)	87,9	16 (0,1)	186,0	14 (0,1)	153,9	9 (0,1)	90,2	120 (0,1)	2.170,3
Espanha	1.152 (8,4)	4.524,7	1.124 (8,3)	4.272,0	1.155 (8,2)	4.050,6	1.062 (7,4)	3.413,0	1.081 (7,6)	3.246,2	1.101 (7,7)	3.155,8	1.013 (7,3)	2.832,7	1.010 (7,3)	2.807,0	1.018 (7,2)	2.836,8	1.057 (6,9)	2.983,6	1.088 (6,1)	3.130,5	974 (6,7)	2.843,5	12.835 (7,4)	36.334,3
Estados Unidos	73 (0,5)	385,3	95 (0,7)	444,7	78 (0,6)	294,3	91 (0,6)	285,7	93 (0,7)	254,8	94 (0,7)	233,4	99 (0,7)	227,7	106 (0,8)	228,5	97 (0,7)	196,4	119 (0,8)	231,8	123 (0,7)	232,9	118 (0,8)	214,9	1.186 (0,7)	2.832,4
França	133 (1,0)	1.368,7	129 (0,9)	1.170,8	119 (0,8)	848,8	128 (0,9)	735,1	96 (0,7)	464,2	117 (0,8)	494,8	125 (0,9)	474,3	117 (0,8)	406,0	119 (0,8)	382,7	119 (0,8)	365,2	173 (1,0)	518,0	126 (0,9)	363,4	1.501 (0,9)	6.003,9
Grécia	55 (0,4)	4.696,8	54 (0,4)	4.368,9	78 (0,6)	5.577,4	79 (0,6)	5.039,9	74 (0,5)	4.202,2	69 (0,5)	3.479,6	53 (0,4)	2.412,9	62 (0,4)	2.650,1	85 (0,6)	3.619,3	67 (0,4)	2.908,0	78 (0,4)	3.457,4	62 (0,4)	2.799,7	816 (0,5)	39.047,7
Guiana	8 (0,1)	1.126,8	9 (0,1)	1.252,6	8 (0,1)	1.078,2	5 (0)	640,6	10 (0,1)	1.209,2	21 (0,1)	2.456,1	14 (0,1)	1.579,2	25 (0,2)	2.691,1	15 (0,1)	1.515,9	19 (0,1)	1.817,3	25 (0,1)	2.347,4	30 (0,2)	2.678,6	189 (0,1)	21.705,4
Haiti	1 (0)	36,0	10 (0,1)	198,1	17 (0,1)	114,6	49 (0,3)	149,7	75 (0,5)	139,2	86 (0,6)	114,2	103 (0,7)	110,4	139 (1,0)	126,3	147 (1,0)	110,3	242 (1,6)	156,5	257 (1,4)	155,4	192 (1,3)	112,9	1.318 (0,8)	1.563,7
Holanda	46 (0,3)	1.510,2	50 (0,4)	1.480,4	52 (0,4)	1.279,4	55 (0,4)	1.132,2	59 (0,4)	1.035,0	64 (0,4)	988,4	63 (0,5)	885,5	57 (0,4)	755,6	54 (0,4)	687,9	53 (0,3)	659,7	68 (0,4)	835,5	55 (0,4)	662,0	676 (0,4)	9.948,5
Hungria	72 (0,5)	11.881,2	67 (0,5)	10.850,2	63 (0,4)	9.820,7	65 (0,5)	9.826,2	62 (0,4)	8.857,1	71 (0,5)	9.752,7	44 (0,3)	6.039,8	54 (0,4)	7.427,8	63 (0,4)	8.955,2	44 (0,3)	6.475,3	54 (0,3)	8.083,8	43 (0,3)	6.544,9	702 (0,4)	96.395,5
Indonésia	17 (0,1)	1.054,6	9 (0,1)	406,9	14 (0,1)	421,1	4 (0)	94,7	8 (0,1)	169,3	7 (0)	143,0	13 (0,1)	260,8	14 (0,1)	277,6	11 (0,1)	215,5	21 (0,1)	408,3	19 (0,1)	367,3	14 (0,1)	267,8	151 (0,1)	3.057,1
Israel	22 (0,2)	2.073,5	11 (0,1)	997,7	21 (0,1)	1.789,5	20 (0,1)	1.596,2	18 (0,1)	1.345,3	19 (0,1)	1.363,5	26 (0,2)	1.811,8	19 (0,1)	1.290,8	27 (0,2)	1.805,4	31 (0,2)	2.046,2	29 (0,2)	1.893,0	29 (0,2)	1.885,0	272 (0,2)	19.232,8
Itália	1.278 (9,3)	4.291,6	1.328 (9,8)	4.349,3	1.350 (9,6)	4.198,7	1.412 (9,9)	4.120,1	1.381 (9,7)	3.782,1	1.365 (9,6)	3.576,7	1.340 (9,7)	3.417,8	1.297 (9,4)	3.264,9	1.338 (9,4)	3.354,8	1.362 (8,9)	3.450,5	1.507 (8,5)	3.902,4	1.220 (8,3)	3.212,1	16.178 (9,3)	41.819,8
Japão	1.936 (14,1)	5.925,9	1.995 (14,6)	6.126,0	2.052 (14,6)	6.289,9	2.019 (14,2)	6.152,6	2.035 (14,3)	6.188,3	2.046 (14,4)	6.246,7	1.904 (13,7)	5.891,5	1.790 (12,9)	5.602,9	1.685 (11,9)	5.337,3	1.825 (11,9)	5.972,7	1.836 (10,3)	6.296,3	1.625 (11,1)	5.794,1	22.748 (13,1)	69.917,5
Jordânia	35 (0,3)	8.046,0	28 (0,2)	6.504,1	23 (0,2)	5.386,4	35 (0,2)	7.918,6	24 (0,2)	5.122,7	34 (0,2)	7.003,1	27 (0,2)	5.373,1	26 (0,2)	4.878,0	32 (0,2)	5.844,7	28 (0,2)	5.204,5	42 (0,2)	8.022,9	22 (0,2)	4.288,5	356 (0,2)	72.064,8
Letônia	11 (0,1)	8.270,7	14 (0,1)	8.536,6	10 (0,1)	4.566,2	7 (0)	2.631,6	11 (0,1)	3.531,3	13 (0,1)	3.651,7	10 (0,1)	2.611,0	9 (0,1)	2.278,5	4 (0)	986,4	6 (0)	1.452,8	9 (0,1)	2.142,9	8 (0,1)	1.851,9	112 (0,1)	30.311,2
Líbano	235 (1,7)	3.528,0	223 (1,6)	3.318,5	289 (2,1)	4.224,5	264 (1,9)	3.798,0	265 (1,9)	3.776,0	306 (2,2)	4.347,5	289 (2,1)	4.100,5	230 (1,7)	3.250,7	284 (2,0)	3.988,5	311 (2,0)	4.385,2	358 (2,0)	5.107,0	252 (1,7)	3.592,3	3.306 (1,9)	46.938,6
Lituânia	99 (0,7)	23.970,9	91 (0,7)	22.864,3	65 (0,5)	17.195,8	63 (0,4)	17.142,9	61 (0,4)	16.968,0	52 (0,4)	14.689,3	55 (0,4)	16.105,4	41 (0,3)	13.162,1	30 (0,2)	10.869,6	45 (0,3)	18.867,9	36 (0,2)	18.181,8	23 (0,2)	13.649,9	661 (0,4)	190.079,1
Marrocos	6 (0)	1.600,0	3 (0)	714,3	12 (0,1)	2.245,1	14 (0,1)	2.054,3	13 (0,1)	1.553,2	11 (0,1)	1.092,4	13 (0,1)	1.090,6	14 (0,1)	1.003,2	11 (0,1)	681,1	18 (0,1)	1.029,2	14 (0,1)	769,2	19 (0,1)	970,9	148 (0,1)	13.460,7
Moçambique	1 (0)	83,8	11 (0,1)	810,9	8 (0,1)	471,6	9 (0,1)	421,2	8 (0,1)	311,2	13 (0,1)	449,0	14 (0,1)	436,3	11 (0,1)	313,3	10 (0,1)	262,4	21 (0,1)	521,4	18 (0,1)	431,5	19 (0,1)	431,3	143 (0,1)	4.685,1
Nigéria	8 (0,1)	583,5	6 (0)	397,0	11 (0,1)	593,3	12 (0,1)	482,2	11 (0,1)	347,2	18 (0,1)	487,2	11 (0,1)	266,8	15 (0,1)	342,6	12 (0,1)	259,5	19 (0,1)	393,1	19 (0,1)	379,2	18 (0,1)	341,1	160 (0,1)	4.093,6
Paraguai	246 (1,8)	1.046,4	218 (1,6)	867,7	226 (1,6)	792,9	232 (1,6)	718,7	272 (1,9)	757,2	279 (2,0)	718,3	263 (1,9)	636,0	302 (2,2)	690,9	326 (2,3)	707,1	358 (2,3)	748,1	464 (2,6)	935,6	350 (2,4)	665,9	3.536 (2,0)	8.818,4
Peru	71 (0,5)	519,8	82 (0,6)	524,3	71 (0,5)	363,8	110 (0,8)	469,0	105 (0,7)	386,8	100 (0,7)	330,2	97 (0,7)	292,7	87 (0,6)	242,0	119 (0,8)	308,0	209 (1,4)	517,3	254 (1,4)	611,0	157 (1,1)	363,6	1.462 (0,8)	4.610,2
Polônia	299 (2,2)	16.537,6	275 (2,0)	14.274,6	245 (1,7)	10.984,1	297 (2,1)	11.320,8	245 (1,7)	8.088,5	238 (1,7)	6.995,9	209 (1,5)	5.656,3	192 (1,4)	4.898,6	217 (1,5)	5.383,9	205 (1,3)	5.147,5	206 (1,2)	5.250,4	178 (1,2)	4.520,1	2.806 (1,6)	79.075,7
Portugal	5.289 (38,5)	4.835,7	5.305 (39,0)	4.871,1	5.475 (38,9)	5.059,0	5.550 (38,9)	5.171,6	5.579 (39,1)	5.292,7	5.430 (38,2)	5.294,2	5.345 (38,6)	5.396,3	5.268 (38,0)	5.538,9	5.307 (37,3)	5.838,6	5.450 (35,6)	6.317,3	5.966 (33,5)	7.354,6	4.945 (33,8)	6.475,9	64.909 (37,3)	64.389,2
Reino Unido	35 (0,3)	726,0	31 (0,2)	553,7	52 (0,4)	724,1	48 (0,3)	546,6	38 (0,3)	370,3	53 (0,4)	456,3	37 (0,3)	291,6	37 (0,3)	275,6	37 (0,3)	262,9	50 (0,3)	343,9	64 (0,4)	430,6	53 (0,4)	346,1	535 (0,3)	4.402,8
República Tcheca	21 (0,2)	6.441,7	20 (0,1)	5.730,7	18 (0,1)	4.265,4	24 (0,2)	4.678,4	29 (0,2)	4.953,0	27 (0,2)	4.208,9	24 (0,2)	3.526,8	21 (0,2)	2.935,0	20 (0,1)	2.631,6	18 (0,1)	2.282,8	12 (0,1)	1.494,4	20 (0,1)	2.428,7	254 (0,1)	38.426,6
Romênia	171 (1,2)	27.099,8	141 (1,0)	18.700,3	158 (1,1)	15.520,6	148 (1,0)	11.526,5	102 (0,7)	6.484,4	110 (0,8)	5.643,9	99 (0,7)	4.395,1	84 (0,6)	3.488,4	90 (0,6)	3.608,7	66 (0,4)	2.637,9	70 (0,4)	2.815,2	61 (0,4)	2.440,5	1.300 (0,7)	61.882,7
Rússia	55 (0,4)	4.293,5	52 (0,4)	3.419,9	64 (0,5)	3.203,2	53 (0,4)	2.161,1	48 (0,3)	1.647,2	41 (0,3)	1.201,6	49 (0,4)	1.277,5	31 (0,2)	740,7	28 (0,2)	620,2	37 (0,2)	775,1	40 (0,2)	785,8	19 (0,1)	337,8	517 (0,3)	14.267,0
Sérvia	67 (0,5)	24.814,8	67 (0,5)	24.907,1	88 (0,6)	33.270,3	41 (0,3)	14.089,3	66 (0,5)	19.969,7	30 (0,2)	8.438,8	36 (0,3)	9.536,4	28 (0,2)	7.282,2	27 (0,2)	7.031,3	37 (0,2)	9.866,7	34 (0,2)	9.497,2	17 (0,1)	4.864,1	538 (0,3)	146.794,0
Síria	84 (0,6)	8.203,1	84 (0,6)	7.850,5	89 (0,6)	7.038,4	86 (0,6)	4.162,6	89 (0,6)	2.834,4	73 (0,5)	1.947,7	89 (0,6)	2.159,9	80 (0,6)	1.875,3	64 (0,5)	1.526,0	93 (0,6)	2.259,7	84 (0,5)	2.085,9	69 (0,5)	1.746,6	984 (0,6)	25.011,1
Suíça	52 (0,4)	2.195,0	59 (0,4)	2.341,3	63 (0,4)	2.241,2	54 (0,4)	1.710,5	63 (0,4)	1.704,8	53 (0,4)	1.233,4	53 (0,4)	1.085,4	74 (0,5)	1.367,1	64 (0,5)	1.090,7	68 (0,4)	1.105,7	68 (0,4)	1.087,7	68 (0,5)	1.055,7	739 (0,4)	16.100,2
Turquia	25 (0,2)	8.771,9	22 (0,2)	6.547,6	22 (0,2)	4.835,2	16 (0,1)	2.644,6	24 (0,2)	3.129,1	15 (0,1)	1.608,6	12 (0,1)	1.087,9	19 (0,1)	1.515,8	18 (0,1)	1.312,4	9 (0,1)	619,8	11 (0,1)	715,7	17 (0,1)	1.012,8	210 (0,1)	20.633,8
Ucrânia	22 (0,2)	3.288,5	16 (0,1)	1.901,4	26 (0,2)	2.125,1	17 (0,1)	1.023,8	19 (0,1)	915,7	20 (0,1)	820,5	16 (0,1)	588,2	18 (0,1)	618,1	12 (0,1)	392,2	15 (0,1)	477,6	13 (0,1)	403,7	15 (0,1)	428,3	209 (0,1)	8.104,7
Uruguai	167 (1,2)	847,3	203 (1,5)	980,8	228 (1,6)	989,9	210 (1,5)	808,8	233 (1,6)	789,0	229 (1,6)	687,3	220 (1,6)	596,5	231 (1,7)	560,2	267 (1,9)	584,1	287 (1,9)	596,6	354 (2,0)	720,0	334 (2,3)	659,0	2.963 (1,7)	8.441,5
Venezuela	10 (0,1)	340,8	15 (0,1)	448,0	8 (0,1)	187,0	13 (0,1)	234,9	15 (0,1)	206,0	16 (0,1)	142,7	54 (0,4)	217,3	140 (1,0)	198,2	335 (2,4)	215,3	451 (2,9)	203,2	731 (4,1)	268,2	731 (5,0)	209,3	2.519 (1,4)	13.968,6
Outros	417 (3,0)	1.558,4	223 (1,6)	665,3	234 (1,7)	483,9	289 (2,0)	444,2	241 (1,7)	293,6	257 (1,8)	265,3	272 (2,0)	250,4	319 (2,3)	267,9	247 (1,7)	192,4	304 (2,0)	226,2	436 (2,4)	315,1	340 (2,3)	234,1	3.579 (2,1)	3.483,4
Total	13.729 (100)	2.778,9	13.619 (100)	2.563,4	14.090 (100)	2.269,2	14.256 (100)	1.952,1	14.259 (100)	1.701,5	14.225 (100)	1.519,1	13.852 (100)	1.341,5	13.850 (100)	1.199,4	14.210 (100)	1.079,2	15.300 (100)	1.065,9	17.797 (100)	1.175,4	14.620 (100)	897,9	173.807 (100)	17.654,4

CM: coeficiente de mortalidade.

O CM no período de 2011 a 2022 foi de 17.654,7 óbitos por 100 mil habitantes. A macrorregião Sudeste do Brasil teve o maior número de óbitos, seguida pela macrorregião Sul. A macrorregião Sudeste aparece novamente em primeiro lugar quando observamos os CM, sucedida pela macrorregião Centro-Oeste. O maior número de óbitos no período ocorreu nos estados de São Paulo, Rio de Janeiro e Paraná, respectivamente. Os maiores CM foram observados nos estados do Rio de Janeiro, São Paulo e Mato Grosso do Sul, nessa ordem (Tabela 3).

**Tabela 3:** Mortalidade de migrantes internacionais residentes no Brasil por Unidade Federativa e macrorregião, 2011 a 2022.

Macrorregião/Unidade Federada	2011			2012			2013			2014			2015			2016			2017			2018			2019			2020			2021			2022			Total		
	n	%	CM	n	%	CM	n	%	CM	n	%	CM	n	%	CM	n	%	CM	n	%	CM	n	%	CM	n	%	CM	n	%	CM	n	%	CM	n	%	CM	n	%	CM
Norte	208	1,5	1.044,3	236	1,7	1.087,9	222	1,6	860,0	236	1,7	768,1	296	2,1	828,9	308	2,2	734,9	350	2,5	631,0	400	2,9	425,8	570	4,0	353,5	758	4,9	359,5	974	5,5	402,9	815	5,6	282,7	5.373	3,1	11.035,3
Acre	5	0	455,0	11	0,1	891,4	9	0,1	601,2	10	0,1	566,6	15	0,1	744,2	13	0,1	582,0	17	0,1	697,0	12	0,1	451,1	10	0,1	338,1	21	0,1	656,1	50	0,3	1.506,0	18	0,1	504,8	191	0,1	8.175,5
Amapá	1	0	130,2	4	0	460,6	8	0,1	765,9	2	0	167,2	3	0	224,6	3	0	203,3	5	0	307,7	3	0	169,2	11	0,1	568,5	9	0,1	438,4	9	0,1	420,8	9	0,1	401,2	67	0	4.321,9
Amazonas	81	0,6	914,7	85	0,6	876,5	74	0,5	625,0	90	0,6	622,0	89	0,6	525,2	99	0,7	506,1	103	0,7	448,3	95	0,7	307,6	158	1,1	330,1	255	1,7	412,6	294	1,7	427,8	233	1,6	302,2	1.656	1,0	7.786,0
Pará	80	0,6	2.012,6	92	0,7	2.148,0	90	0,6	1.810,7	74	0,5	1.292,2	118	0,8	1.826,8	103	0,7	1.431,1	101	0,7	1.261,2	103	0,7	1.146,9	114	0,8	1.130,5	138	0,9	1.277,7	147	0,8	1.303,9	104	0,7	871,9	1.264	0,7	16.625,0
Rondônia	18	0,1	521,0	24	0,2	640,3	22	0,2	505,0	38	0,3	754,2	43	0,3	758,4	47	0,3	763,7	59	0,4	893,4	50	0,4	682,8	63	0,4	730,6	84	0,5	863,3	95	0,5	902,9	77	0,5	660,4	620	0,4	9.719,4
Roraima	16	0,1	1.300,8	17	0,1	1.328,1	16	0,1	1.136,8	15	0,1	884,4	19	0,1	816,0	31	0,2	734,3	54	0,4	428,1	130	0,9	317,3	211	1,5	238,9	236	1,5	193,9	365	2,1	253,2	369	2,5	204,9	1.479	0,9	17.570,5
Tocantins	7	0,1	1.308,4	3	0	515,9	3	0	430,1	7	0	838,3	9	0,1	943,4	12	0,1	1.125,2	11	0,1	915,5	7	0,1	520,8	3	0	204,2	15	0,1	970,6	14	0,1	872,0	5	0	296,4	96	0,1	8.465,6
Nordeste	334	2,4	1.047,7	371	2,7	1.031,1	355	2,5	778,9	422	3,0	737,8	382	2,7	552,7	434	3,1	545,0	448	3,2	505,4	492	3,6	505,1	523	3,7	494,3	579	3,8	520,3	671	3,8	583,0	586	4,0	488,3	5.597	3,2	6.652,2
Alagoas	10	0,1	1.042,8	12	0,1	1.116,3	10	0,1	740,7	14	0,1	838,3	4	0	202,2	8	0,1	357,8	12	0,1	482,6	12	0,1	434,6	24	0,2	792,2	18	0,1	560,0	12	0,1	356,0	17	0,1	475,1	153	0,1	6.479,6
Bahia	137	1,0	1.107,5	166	1,2	1.219,6	152	1,1	930,5	191	1,3	990,1	151	1,1	686,7	164	1,2	671,1	184	1,3	684,6	204	1,5	696,1	202	1,4	640,5	216	1,4	654,3	250	1,4	732,9	209	1,4	588,9	2.226	1,3	8.675,9
Ceará	44	0,3	734,6	53	0,4	756,3	57	0,4	610,9	64	0,4	526,5	67	0,5	434,6	73	0,5	396,1	63	0,5	305,1	74	0,5	328,8	80	0,6	330,3	90	0,6	355,3	121	0,7	464,3	99	0,7	364,3	885	0,5	4.529,8
Maranhão	22	0,2	1.305,6	13	0,1	667,7	21	0,1	808,5	9	0,1	270,1	14	0,1	346,0	17	0,1	353,5	20	0,1	363,3	18	0,1	291,0	27	0,2	394,2	27	0,2	373,0	47	0,3	626,9	36	0,2	461,0	271	0,2	5.255,0
Paraíba	15	0,1	856,2	14	0,1	713,7	16	0,1	639,0	25	0,2	764,6	19	0,1	460,9	18	0,1	375,5	25	0,2	475,6	24	0,2	420,4	25	0,2	404,8	28	0,2	431,4	35	0,2	522,0	32	0,2	457,8	276	0,2	5.492,8
Pernambuco	53	0,4	1.027,9	64	0,5	1.115,0	63	0,4	871,5	70	0,5	746,9	69	0,5	585,7	83	0,6	600,9	94	0,7	608,1	100	0,7	583,2	99	0,7	527,1	118	0,8	596,8	137	0,8	671,6	110	0,8	518,7	1.060	0,6	7.242,9
Piauí	4	0	923,8	3	0	596,4	3	0	423,4	6	0	601,8	4	0	319,9	9	0,1	616,9	6	0	342,7	8	0,1	384,0	8	0,1	329,9	10	0,1	373,7	6	0	213,6	10	0,1	340,0	77	0	4.797,5
Rio Grande do Norte	31	0,2	1.090,4	36	0,3	1.083,4	19	0,1	427,0	29	0,2	504,8	41	0,3	595,2	48	0,3	615,8	39	0,3	454,1	37	0,3	396,3	43	0,3	428,1	58	0,4	547,1	47	0,3	424,1	54	0,4	465,0	482	0,3	5.884,1
Sergipe	18	0,1	2.608,7	10	0,1	1.232,3	14	0,1	1.302,3	14	0,1	1.023,0	13	0,1	788,8	14	0,1	749,5	5	0	241,7	15	0,1	631,4	15	0,1	547,0	14	0,1	474,6	16	0,1	522,9	19	0,1	594,6	167	0,1	8.484,7
Centro-Oeste	454	3,3	1.780,4	407	3,0	1.501,3	459	3,3	1.410,5	477	3,3	1.180,6	517	3,6	1.082,9	492	3,5	896,5	450	3,3	712,9	480	3,5	671,9	591	4,2	733,9	627	4,1	712,6	1.074	6,0	1.146,5	749	5,1	742,2	6.777	3,9	11.486,0
Distrito Federal	114	0,8	1.720,2	97	0,7	1.355,2	92	0,7	969,3	129	0,9	995,1	134	0,9	857,6	95	0,7	506,7	101	0,7	432,2	115	0,8	426,2	127	0,9	435,5	166	1,1	541,2	170	1,0	534,3	98	0,7	294,4	1.438	0,8	6.829,0
Goiás	70	0,5	1.108,8	59	0,4	872,6	94	0,7	1.197,9	99	0,7	1.072,8	74	0,5	692,4	94	0,7	782,3	74	0,5	556,3	80	0,6	538,4	130	0,9	775,2	119	0,8	650,6	431	2,4	2.240,4	277	1,9	1.367,8	1.601	0,9	12.647,1
Mato Grosso	66	0,5	1.965,5	57	0,4	1.568,5	52	0,4	1.105,7	64	0,4	1.007,1	73	0,5	899,2	79	0,6	819,9	65	0,5	596,4	84	0,6	668,6	76	0,5	503,3	102	0,7	592,3	123	0,7	658,7	91	0,6	443,4	932	0,5	9.077,6
Mato Grosso do Sul	204	1,5	2.216,9	194	1,4	2.030,0	221	1,6	2.104,6	185	1,3	1.560,4	236	1,7	1.772,8	224	1,6	1.547,0	210	1,5	1.349,7	201	1,5	1.179,6	258	1,8	1.323,8	240	1,6	1.100,7	350	2,0	1.461,2	283	1,9	1.053,8	2.806	1,6	18.683,0
Sudeste	11.329	82,5	3.287,5	11.244	82,6	3.053,4	11.580	82,1	2.749,1	11.677	81,9	2.431,9	11.618	81,5	2.172,6	11.460	80,6	1.966,8	11.207	81,0	1.795,0	10.935	79,0	1.647,9	10.926	76,9	1.550,0	11.647	76,0	1.592,4	13.094	73,6	1.754,9	10.740	73,5	1.396,4	137.457	79,1	22.776,3
Espírito Santo	66	0,5	1.433,5	72	0,5	1.433,0	60	0,4	932,0	60	0,4	719,6	56	0,4	573,5	52	0,4	484,9	56	0,4	487,5	62	0,4	510,0	54	0,4	419,5	65	0,4	484,9	90	0,5	653,1	72	0,5	505,8	765	0,4	6.888,5
Minas Gerais	218	1,6	1.142,6	317	2,3	1.537,1	318	2,3	1.299,7	323	2,3	1.100,5	351	2,5	1.023,4	329	2,3	845,5	324	2,3	748,8	345	2,5	723,5	355	2,5	672,7	370	2,4	653,1	468	2,6	792,5	362	2,5	581,9	4.080	2,3	9.929,4
Rio de Janeiro	3.120	22,7	3.598,7	3.148	23,1	3.398,1	3.157	22,4	2.985,9	3.194	22,4	2.658,4	3.266	22,9	2.447,5	3.177	22,3	2.194,3	3.102	22,4	2.024,1	2.975	21,5	1.867,4	3.108	21,9	1.886,4	3.246	21,2	1.937,7	3.518	19,8	2.086,2	2.764	18,9	1.611,0	37.775	21,7	25.349,2
São Paulo	7.925	57,7	3.383,5	7.707	56,6	3.083,4	8.045	57,1	2.826,9	8.100	56,8	2.513,1	7.945	55,7	2.224,0	7.902	55,5	2.035,3	7.725	55,8	1.855,5	7.553	54,5	1.699,5	7.409	52,1	1.561,5	7.966	52,0	1.613,1	9.018	50,7	1.786,9	7.542	51,6	1.447,4	94.837	54,6	23.574,1
Sul	1.405	10,2	1.954,0	1.362	10,0	1.757,3	1.483	10,5	1.580,7	1.450	10,2	1.226,0	1.445	10,1	996,5	1.533	10,8	906,0	1.388	10,0	727,8	1.543	11,1	713,2	1.599	11,3	636,1	1.705	11,1	607,8	1.966	11,1	646,9	1.724	11,8	513,3	18.603	10,7	10.337,1
Paraná	905	6,6	2.883,5	822	6,0	2.445,4	843	6,0	2.123,6	870	6,1	1.816,9	819	5,7	1.457,8	874	6,1	1.372,7	805	5,8	1.141,8	847	6,1	1.079,8	862	6,1	961,8	919	6,0	924,3	1.013	5,7	937,5	848	5,8	706,9	10.427	6,0	15.542,7
Rio Grande do Sul	328	2,4	1.236,5	355	2,6	1.249,1	464	3,3	1.374,1	381	2,7	914,1	406	2,8	804,7	437	3,1	746,7	358	2,6	544,8	398	2,9	532,5	452	3,2	521,6	471	3,1	492,7	537	3,0	528,8	505	3,5	462,3	5.092	2,9	8.197,6
Santa Catarina	172	1,3	1.229,2	185	1,4	1.195,9	176	1,2	864,8	199	1,4	693,3	220	1,5	573,4	222	1,6	472,2	225	1,6	412,7	298	2,2	471,7	285	2,0	379,4	315	2,1	368,4	416	2,3	441,1	371	2,5	347,9	3.084	1,8	6.075,4
Total	13.730	100	2.779,1	13.620	100	2.563,6	14.099	100	2.270,6	14.262	100	1.952,9	14.258	100	1.701,4	14.227	100	1.519,4	13.843	100	1.340,6	13.850	100	1.199,4	14.209	100	1.079,1	15.316	100	1.067,1	17.779	100	1.174,2	14.614	100	897,6	173.807	100	17.654,7

CM: coeficiente de mortalidade.

Em relação aos capítulos da CID-10, houve mudança no perfil de óbitos devido à pandemia de covid-19 (Tabela 4). Em quase todos os anos, a maioria dos óbitos ocorreu de forma mais frequente por “doenças do aparelho circulatório” (Capítulo IX); porém, em 2021 esse perfil mudou, com “algumas doenças infecciosas e parasitárias” (Capítulo I), sendo o capítulo da CID-10 mais frequente. Ao longo do período, os capítulos de “neoplasias” (Capítulo II), “doenças do aparelho respiratório” (Capítulo X) e “causas externas de mobilidade e mortalidade” (Capítulo XX) também aparecem entre as maiores frequências absolutas e relativas (Tabela 4). Na análise de causas básicas mais frequentes no período, infarto agudo do miocárdio não especificado (I21.9), pneumonia não especificada (J18.9) e infecção por coronavírus de localização não especificada (B34.2) são os mais frequentes (Tabela 5).

**Tabela 4:** Mortalidade de migrantes internacionais residentes no Brasil por capítulo da CID-10, 2011 a 2022.

Número do capítulo	Capítulo CID-10 - nome do capítulo	2011		2012		2013		2014		2015		2016		2017		2018		2019		2020		2021		2022		Total	
		n	%	n	%	n	%	n	%	n	%	n	%	n	%	n	%	n	%	n	%	n	%	n	%	n	%
I	Algumas doenças infecciosas e parasitárias	368	2,7	405	3,0	428	3,0	484	3,4	522	3,7	483	3,4	478	3,5	513	3,7	524	3,7	2.894	18,9	4.460	25,1	1.516	10,4	13.075	7,5
II	Neoplasias (tumores)	2.619	19,1	2.614	19,2	2.669	18,9	2.645	18,5	2.684	18,8	2.700	19,0	2.605	18,8	2.656	19,2	2.572	18,1	2.371	15,5	2.544	14,3	2.434	16,7	31.113	17,9
III	Doenças do sangue e dos órgãos hematopoiéticos e alguns transtornos imunitários	85	0,6	92	0,7	85	0,6	100	0,7	83	0,6	101	0,7	78	0,6	99	0,7	92	0,6	101	0,7	99	0,6	104	0,7	1.119	0,6
IV	Doenças endócrinas nutricionais e metabólicas	809	5,9	747	5,5	757	5,4	766	5,4	752	5,3	699	4,9	702	5,1	676	4,9	747	5,3	722	4,7	788	4,4	680	4,7	8.845	5,1
V	Transtornos mentais e comportamentais	198	1,4	164	1,2	198	1,4	215	1,5	177	1,2	166	1,2	153	1,1	196	1,4	193	1,4	168	1,1	192	1,1	191	1,3	2.211	1,3
VI	Doenças do sistema nervoso	584	4,3	648	4,8	712	5,1	747	5,2	771	5,4	784	5,5	763	5,5	746	5,4	789	5,6	702	4,6	760	4,3	770	5,3	8.776	5,0
VII	Doenças do olho e anexos	1	0	1	0	0	0	0	0	0	0	0	0	0	0	0	0	0	0	0	0	0	0	0	0	2	0
VIII	Doenças do ouvido e da apófise mastoide	0	0	2	0	2	0	0	0	0	0	1	0	1	0	2	0	2	0	0	0	0	0	2	0	12	0
IX	Doenças do aparelho circulatório	4.359	31,7	4.264	31,3	4.393	31,2	4.296	30,1	4.164	29,2	4.252	29,9	4.080	29,5	3.881	28,0	4.010	28,2	3.579	23,4	3.814	21,5	3.615	24,7	48.707	28,0
X	Doenças do aparelho respiratório	2.252	16,4	2.151	15,8	2.327	16,5	2.342	16,4	2.400	16,8	2.261	15,9	2.246	16,2	2.115	15,3	2.260	15,9	1.956	12,8	1.744	9,8	1.982	13,6	26.036	15,0
XI	Doenças do aparelho digestivo	628	4,6	629	4,6	604	4,3	635	4,5	602	4,2	640	4,5	636	4,6	598	4,3	654	4,6	528	3,4	649	3,7	645	4,4	7.448	4,3
XII	Doenças da pele e do tecido subcutâneo	54	0,4	76	0,6	67	0,5	76	0,5	88	0,6	83	0,6	85	0,6	93	0,7	112	0,8	64	0,4	98	0,6	106	0,7	1.002	0,6
XIII	Doenças do sistema osteomuscular e do tecido conjuntivo	73	0,5	81	0,6	73	0,5	93	0,7	90	0,6	76	0,5	92	0,7	85	0,6	93	0,7	67	0,4	86	0,5	72	0,5	981	0,6
XIV	Doenças do aparelho geniturinário	492	3,6	495	3,6	590	4,2	646	4,5	658	4,6	668	4,7	641	4,6	778	5,6	742	5,2	645	4,2	762	4,3	770	5,3	7.887	4,5
XV	Gravidez parto e puerpério	6	0	4	0	0	0	13	0,1	7	0	14	0,1	10	0,1	22	0,2	18	0,1	26	0,2	34	0,2	22	0,2	176	0,1
XVI	Algumas afecções originadas no período perinatal	4	0	4	0	1	0	3	0	1	0	1	0	4	0	2	0	1	0	3	0	14	0,1	19	0,1	57	0
XVII	Malformações congênitas, deformidades e anomalias cromossômicas	12	0,1	14	0,1	9	0,1	11	0,1	7	0	19	0,1	12	0,1	15	0,1	20	0,1	15	0,1	26	0,1	13	0,1	173	0,1
XVIII	Sintomas, sinais e achados anormais de exames clínicos e de laboratório	509	3,7	499	3,7	476	3,4	467	3,3	455	3,2	518	3,6	493	3,6	526	3,8	550	3,9	663	4,3	758	4,3	697	4,8	6.611	3,8
XX	Causas externas de morbidade e mortalidade	677	4,9	730	5,4	708	5,0	723	5,1	797	5,6	761	5,3	764	5,5	847	6,1	830	5,8	812	5,3	951	5,3	976	6,7	9.576	5,5
	Total	13.730	100	13.620	100	14.099	100	14.262	100	14.258	100	14.227	100	13.843	100	13.850	100	14.209	100	15.316	100	17.779	100	14.614	100	173.807	100

CID: Classificação Internacional de Doenças.

**Tabela 5:** Mortalidade de migrantes internacionais residentes no Brasil por causa básica de óbito, 2011 a 2022.

CID-10	Causa básica	2011		2012		2013		2014		2015		2016		2017		2018		2019		2020		2021		2022		Total	
		n	%	n	%	n	%	n	%	n	%	n	%	n	%	n	%	n	%	n	%	n	%	n	%	n	%
A419	Septicemia não especificada	170	1,2	209	1,5	220	1,6	258	1,8	276	1,9	249	1,8	256	1,8	240	1,7	277	1,9	205	1,3	273	1,5	296	2,0	2.929	1,7
B342	Infecção por coronavírus de localização não especificada	0	0	0	0	0	0	0	0	0	0	0	0	0	0	0	0	0	0	2.431	15,9	3.842	21,6	904	6,2	7.177	4,1
C169	Neoplasia maligna do estômago, não especificado	194	1,4	188	1,4	200	1,4	209	1,5	170	1,2	183	1,3	163	1,2	175	1,3	152	1,1	128	0,8	129	0,7	104	0,7	1.995	1,1
C189	Neoplasia maligna do cólon, não especificado	223	1,6	224	1,6	220	1,6	217	1,5	226	1,6	194	1,4	201	1,5	229	1,7	202	1,4	169	1,1	156	0,9	172	1,2	2.433	1,4
C20	Neoplasia maligna do reto	77	0,6	79	0,6	95	0,7	97	0,7	93	0,7	98	0,7	91	0,7	79	0,6	58	0,4	58	0,4	69	0,4	82	0,6	976	0,6
C229	Neoplasia maligna do fígado, não especificado	60	0,4	53	0,4	66	0,5	64	0,4	58	0,4	70	0,5	81	0,6	58	0,4	77	0,5	64	0,4	70	0,4	58	0,4	779	0,4
C259	Neoplasia maligna do pâncreas, não especificado	131	1,0	138	1,0	145	1,0	131	0,9	167	1,2	142	1,0	132	1,0	159	1,1	156	1,1	156	1,0	148	0,8	141	1,0	1.746	1,0
C349	Neoplasia maligna dos brônquios ou pulmões, não especificados	326	2,4	316	2,3	351	2,5	323	2,3	332	2,3	352	2,5	329	2,4	360	2,6	311	2,2	293	1,9	299	1,7	271	1,9	3.863	2,2
C509	Neoplasia maligna da mama, não especificada	159	1,2	172	1,3	162	1,1	147	1,0	162	1,1	148	1,0	150	1,1	154	1,1	158	1,1	139	0,9	144	0,8	156	1,1	1.851	1,1
C61	Neoplasia maligna da próstata	227	1,7	256	1,9	233	1,7	230	1,6	204	1,4	230	1,6	238	1,7	194	1,4	217	1,5	194	1,3	201	1,1	183	1,3	2.607	1,5
C679	Neoplasia maligna da bexiga, sem outras especificações	90	0,7	79	0,6	89	0,6	97	0,7	106	0,7	86	0,6	99	0,7	107	0,8	103	0,7	93	0,6	105	0,6	91	0,6	1.145	0,7
C80	Neoplasia maligna, sem especificação de localização	72	0,5	62	0,5	80	0,6	83	0,6	82	0,6	73	0,5	64	0,5	62	0,4	57	0,4	58	0,4	59	0,3	61	0,4	813	0,5
E142	Diabetes mellitus não especificado - com complicações renais	110	0,8	101	0,7	101	0,7	91	0,6	102	0,7	98	0,7	89	0,6	88	0,6	73	0,5	77	0,5	113	0,6	81	0,6	1.124	0,6
E149	Diabetes mellitus não especificado - sem complicações	320	2,3	314	2,3	309	2,2	308	2,2	318	2,2	275	1,9	273	2,0	260	1,9	288	2,0	287	1,9	300	1,7	273	1,9	3.525	2,0
F03	Demência não especificada	159	1,2	127	0,9	164	1,2	172	1,2	137	1,0	131	0,9	122	0,9	149	1,1	145	1,0	117	0,8	145	0,8	149	1,0	1.717	1,0
G20	Doença de Parkinson	76	0,6	99	0,7	93	0,7	108	0,8	112	0,8	116	0,8	102	0,7	117	0,8	106	0,7	99	0,6	112	0,6	109	0,7	1.249	0,7
G309	Doença de Alzheimer não especificada	404	2,9	418	3,1	469	3,3	492	3,4	516	3,6	517	3,6	507	3,7	473	3,4	513	3,6	440	2,9	469	2,6	467	3,2	5.685	3,3
I10	Hipertensão essencial (primária)	197	1,4	197	1,4	211	1,5	231	1,6	221	1,6	202	1,4	207	1,5	198	1,4	222	1,6	294	1,9	304	1,7	242	1,7	2.726	1,6
I110	Doença cardíaca hipertensiva com insuficiência cardíaca (congestiva)	127	0,9	109	0,8	109	0,8	117	0,8	111	0,8	101	0,7	126	0,9	93	0,7	114	0,8	126	0,8	117	0,7	80	0,5	1.330	0,8
I219	Infarto agudo do miocárdio não especificado	1.072	7,8	1.072	7,9	1.074	7,6	1.100	7,7	1.129	7,9	1.035	7,3	1.030	7,4	951	6,9	1.026	7,2	931	6,1	900	5,1	821	5,6	12.141	7,0
I251	Doença aterosclerótica do coração	166	1,2	150	1,1	154	1,1	160	1,1	136	1,0	155	1,1	136	1,0	145	1,0	149	1,0	86	0,6	78	0,4	94	0,6	1.609	0,9
I255	Miocardiopatia isquêmica	116	0,8	113	0,8	106	0,8	99	0,7	100	0,7	93	0,7	96	0,7	93	0,7	118	0,8	66	0,4	74	0,4	61	0,4	1.135	0,7
I259	Doença isquêmica crônica do coração não especificada	60	0,4	53	0,4	80	0,6	62	0,4	59	0,4	72	0,5	82	0,6	70	0,5	46	0,3	47	0,3	67	0,4	69	0,5	767	0,4
I269	Embolia pulmonar sem menção de cor pulmonale agudo	110	0,8	99	0,7	110	0,8	113	0,8	118	0,8	131	0,9	114	0,8	102	0,7	89	0,6	78	0,5	104	0,6	121	0,8	1.289	0,7
I420	Cardiomiopatia dilatada	110	0,8	114	0,8	131	0,9	89	0,6	73	0,5	98	0,7	79	0,6	70	0,5	68	0,5	37	0,2	39	0,2	58	0,4	966	0,6
I48	Flutter e fibrilação atrial	64	0,5	60	0,4	77	0,5	82	0,6	63	0,4	70	0,5	79	0,6	92	0,7	101	0,7	70	0,5	87	0,5	93	0,6	938	0,5
I499	Arritmia cardíaca não especificada	52	0,4	54	0,4	63	0,4	86	0,6	78	0,5	79	0,6	66	0,5	47	0,3	70	0,5	48	0,3	75	0,4	41	0,3	759	0,4
I500	Insuficiência cardíaca congestiva	180	1,3	168	1,2	172	1,2	151	1,1	164	1,2	172	1,2	145	1,0	140	1,0	154	1,1	115	0,8	132	0,7	121	0,8	1.814	1,0
I509	Insuficiência cardíaca não especificada	130	0,9	124	0,9	144	1,0	138	1,0	156	1,1	157	1,1	166	1,2	144	1,0	158	1,1	172	1,1	189	1,1	212	1,5	1.890	1,1
I619	Hemorragia intracerebral não especificada	154	1,1	173	1,3	175	1,2	184	1,3	145	1,0	172	1,2	137	1,0	152	1,1	152	1,1	141	0,9	157	0,9	150	1,0	1.892	1,1
I639	Infarto cerebral não especificado	118	0,9	135	1,0	149	1,1	132	0,9	121	0,8	131	0,9	137	1,0	145	1,0	154	1,1	133	0,9	129	0,7	105	0,7	1.589	0,9
I64	Acidente vascular cerebral, não especificado como hemorrágico ou isquêmico	418	3,0	362	2,7	349	2,5	327	2,3	334	2,3	317	2,2	281	2,0	242	1,7	237	1,7	230	1,5	258	1,5	227	1,6	3.582	2,1
I678	Outras doenças cerebrovasculares especificadas	139	1,0	142	1,0	126	0,9	140	1,0	105	0,7	134	0,9	100	0,7	93	0,7	90	0,6	83	0,5	89	0,5	84	0,6	1.325	0,8
I694	Sequelas de acidente vascular cerebral não especificado como hemorrágico ou isquêmico	193	1,4	189	1,4	176	1,2	139	1,0	156	1,1	173	1,2	127	0,9	121	0,9	103	0,7	95	0,6	93	0,5	95	0,7	1.660	1,0
J159	Pneumonia bacteriana não especificada	94	0,7	64	0,5	73	0,5	92	0,6	89	0,6	113	0,8	132	1,0	169	1,2	197	1,4	186	1,2	238	1,3	237	1,6	1.684	1,0
J180	Broncopneumonia não especificada	489	3,6	489	3,6	499	3,5	491	3,4	515	3,6	393	2,8	397	2,9	377	2,7	360	2,5	181	1,2	206	1,2	293	2,0	4.690	2,7
J189	Pneumonia não especificada	731	5,3	747	5,5	838	5,9	868	6,1	892	6,3	930	6,5	883	6,4	753	5,4	821	5,8	462	3,0	430	2,4	587	4,0	8.942	5,1
J440	Doença pulmonar obstrutiva crônica com infecção respiratória aguda do trato respiratório inferior	276	2,0	246	1,8	262	1,9	240	1,7	270	1,9	216	1,5	227	1,6	198	1,4	221	1,6	114	0,7	119	0,7	150	1,0	2.539	1,5
J449	Doença pulmonar obstrutiva crônica não especificada	187	1,4	173	1,3	144	1,0	146	1,0	166	1,2	156	1,1	138	1,0	135	1,0	154	1,1	111	0,7	98	0,6	130	0,9	1.738	1,0
J690	Pneumonite devida a alimento ou vômito	78	0,6	87	0,6	104	0,7	99	0,7	86	0,6	96	0,7	104	0,8	100	0,7	120	0,8	97	0,6	163	0,9	131	0,9	1.265	0,7
J988	Outros transtornos respiratórios especificados	28	0,2	21	0,2	29	0,2	35	0,2	20	0,1	17	0,1	18	0,1	12	0,1	18	0,1	434	2,8	110	0,6	16	0,1	758	0,4
K746	Outras formas de cirrose hepática e as não especificadas	57	0,4	59	0,4	62	0,4	51	0,4	69	0,5	57	0,4	53	0,4	61	0,4	57	0,4	46	0,3	59	0,3	65	0,4	696	0,4
N189	Insuficiência renal crônica não especificada	62	0,5	59	0,4	65	0,5	73	0,5	72	0,5	68	0,5	80	0,6	77	0,6	72	0,5	67	0,4	77	0,4	75	0,5	847	0,5
N390	Infecção do trato urinário de localização não especificada	274	2,0	292	2,1	354	2,5	408	2,9	405	2,8	431	3,0	401	2,9	469	3,4	452	3,2	381	2,5	449	2,5	455	3,1	4.771	2,7
R54	Senilidade	94	0,7	92	0,7	95	0,7	103	0,7	80	0,6	89	0,6	71	0,5	82	0,6	61	0,4	76	0,5	85	0,5	111	0,8	1.039	0,6
R98	Morte sem assistência	78	0,6	72	0,5	62	0,4	56	0,4	48	0,3	63	0,4	55	0,4	49	0,4	56	0,4	64	0,4	54	0,3	51	0,3	708	0,4
R99	Outras causas mal definidas e as não especificadas de mortalidade	230	1,7	228	1,7	205	1,5	194	1,4	218	1,5	250	1,8	249	1,8	295	2,1	322	2,3	400	2,6	481	2,7	407	2,8	3.479	2,0
W189	Outras quedas no mesmo nível - local não especificado	58	0,4	48	0,4	60	0,4	65	0,5	88	0,6	79	0,6	59	0,4	66	0,5	70	0,5	40	0,3	53	0,3	43	0,3	729	0,4
Y349	Fatos ou eventos não especificados e intenção não determinada - local não especificado	61	0,4	67	0,5	53	0,4	52	0,4	61	0,4	49	0,3	69	0,5	78	0,6	89	0,6	81	0,5	104	0,6	105	0,7	869	0,5
-	Outros	4.729	34,4	4.727	34,7	4.791	34,0	4.912	34,4	4.849	34,0	4.966	34,9	4.902	35,4	5.127	37,0	5.195	36,6	4.816	31,4	5.527	31,1	5.486	37,5	60.027	34,5
-	Total	13.730	100	13.620	100	14.099	100	14.262	100	14.258	100	14.227	100	13.843	100	13.850	100	14.209	100	15.316	100	17.779	100	14.614	100	173.807	100

Por fim, ao analisar os óbitos de pessoas oriundas de países que enfrentam ou enfrentaram crises migratórias e que têm populações de migrantes em situação de vulnerabilidade residindo no Brasil nos últimos anos, observamos que, com exceção da Síria, há presença de causas desconhecidas, causas evitáveis e violência entre as 10 principais causas de básicas de morte de cada país, a saber: Angola (causas mal definidas e neoplasia de mama), Haiti (causas mal definidas e HIV/aids), Síria (mesmas causas de óbitos de migrantes internacionais vindos de países europeus) e Venezuela (causas mal definidas, neoplasia do colo do útero, neoplasia de mama e violência por armas de fogo e por meio de objetos cortantes e penetrantes). A covid-19 aparece entre as 10 principais causas de óbitos entre migrantes de todas essas naturalidades.

## DISCUSSÃO

Este estudo utilizou pela primeira vez os dados secundários disponíveis para elaboração de CM de migrantes internacionais no Brasil. Também apresentou de forma inédita a mortalidade geral desse grupo populacional por características demográficas, causas de morte e distribuição geográfica desses óbitos. Os dados indicam um elevado número de óbitos em 2021, refletindo o impacto da pandemia de covid-19, que alterou significativamente o perfil de mortalidade dos migrantes internacionais residentes no Brasil.

A predominância de óbitos entre os homens, idosos, com raça/cor da pele branca, viúvos ou casados é consistente com a literatura nacional e internacional existente sobre mortalidade geral, sugerindo que há vulnerabilidade às doenças crônicas não transmissíveis e condições de saúde associadas ao envelhecimento^4,8,17^. A maior frequência de óbitos entre migrantes de países como Portugal, Japão e Itália pode ser explicada pelas ondas migratórias históricas do início do século XX, resultando em uma população migrante envelhecida que atualmente reside no Brasil^3,18^.

Os CM mais elevados entre migrantes nascidos na Lituânia, Sérvia e Hungria, entre outros, podem ser atribuídos tanto a denominadores com populações pequenas, inflando artificialmente os CM, como também a condições de saúde pré-existentes e acesso desigual aos serviços de saúde^19^. A concentração dos óbitos nas macrorregiões Sudeste e Sul, particularmente nos estados de São Paulo, Rio de Janeiro e Paraná, é coerente com a distribuição demográfica dos migrantes no país, em regiões mais favoráveis à busca por trabalho e renda^22^.

A pandemia de covid-19 teve um impacto significativo no perfil de mortalidade dos migrantes, com “doenças do aparelho circulatório” sendo superadas por “algumas doenças infecciosas e parasitárias” como capítulo da causa de morte mais frequente em 2021, assim como aconteceu com a população brasileira de forma geral^23^. Isso demonstra que essas populações são afetadas pelas crises de emergências em saúde pública e destaca a necessidade de políticas específicas de saúde, principalmente de prioridade na fila de vacinação para populações de migrantes internacionais em situação de vulnerabilidade^6^.

As causas básicas de óbito mais frequentes fazem sentido com o perfil sociodemográfico desses óbitos, pois são causas básicas de óbitos que no geral ocorrem majoritariamente em idosos^24^. Observa-se também uma elevada prevalência de causas de morte evitáveis e de violência entre migrantes internacionais nascidos em países que estavam ou estão atualmente em crise econômica e sofrendo com emergências em saúde pública ou emergências humanitárias (surtos, epidemias, desastres, desassistências, mudanças climáticas, conflitos e guerras)^4,6,25^. Esses dados sugerem que migrantes de tais regiões enfrentam barreiras adicionais no acesso à saúde e podem estar expostos a condições de vida mais precárias, o que contribui para essas frequências maiores de óbitos por causas preveníveis.

Um estudo realizado sobre a mortalidade de migrantes internos e internacionais no Brasil, utilizando dados da coorte de 100 milhões de brasileiros do Centro de Integração de Dados e Conhecimentos para Saúde da Fundação Oswaldo Cruz (Cidacs/Fiocruz), chegou a resultados e conclusões semelhantes as deste estudo, principalmente sobre a necessidade de políticas públicas de saúde que de fato englobem as diferentes características socioeconômicas e especificidades dos migrantes internacionais que residem no Brasil^17^.

Embora o Brasil tenha avançado em termos de políticas públicas para migrantes internacionais, como a Lei de Migração de 2017 e o retorno ao Pacto Global para Migração Segura, Ordenada e Regular e a criação do Grupo de Trabalho para estabelecimento da Política Nacional de Saúde das Populações Migrantes, Refugiadas e Apátridas, ainda existem desafios significativos^28^. A invisibilidade dos migrantes internacionais nas estatísticas de saúde, devido à falta de dados completos e à ausência de preenchimento obrigatório de variáveis importantes em sistemas de vigilância em saúde, dificulta a implementação de estratégias eficazes de saúde pública.

O presente estudo apresenta limitações. O Censo de 2022 do Instituto Brasileiro de Geografia e Estatística não teve os resultados liberados sobre a população migrante até o final da execução desta pesquisa, o que inviabilizou estimativas mais precisas dos denominadores para cálculo dos CM. Os denominadores não consideraram, por exemplo, migrantes internacionais indocumentados no Brasil ou migrantes internacionais que emigraram durante o período do estudo. Não foi possível realizar uma comparação dos CM de migrantes internacionais residentes no Brasil com os CM de brasileiros residentes no Brasil, pois acreditamos que essas populações tenham estrutura etária diferentes entre si (cada país de nascimento possui uma estrutura etária distinta) e diferentes da população brasileira, o que não permite o cálculo de CM padronizados, que viabilizaria tal comparação.

Não foi possível realizar interpolação linear para as populações de migrantes por municípios, devido ao deslocamento dos endereços de residências que eles fazem constantemente entre cidades próximas, o que impossibilitou que a análise fosse feita por mesorregiões. Tentamos minimizar essa limitação ao elaborá-las por macrorregiões e UF, pois são unidades de análises maiores e que diminuem a possibilidade de viés.

Apesar das limitações, este estudo reforça a necessidade urgente de inclusão dos migrantes internacionais nas políticas de saúde pública, reconhecendo-os como uma população em situação de vulnerabilidade e prioritária. Políticas específicas que garantam equidade no acesso a serviços de saúde, medicamentos e vacinas são essenciais para melhorar as condições de saúde dessa população a longo prazo. É fundamental aprimorar a coleta de dados e a integração dos migrantes nos sistemas de informação em saúde, para permitir uma melhor preparação, vigilância e resposta às suas necessidades de saúde.

## Footnotes

a As populações calculadas de 2011 a 2022 e utilizadas como denominadores podem ser acessadas no seguinte link: https://github.com/joaorobertobr/denominadores_migrantes.
